# Training Medical Specialists to Communicate Better with Patients with Medically Unexplained Physical Symptoms (MUPS). A Randomized, Controlled Trial

**DOI:** 10.1371/journal.pone.0138342

**Published:** 2015-09-18

**Authors:** Anne Weiland, Annette H. Blankenstein, Jan L. C. M. Van Saase, Henk T. Van der Molen, Mariël E. Jacobs, Dineke C. Abels, Nedim Köse, Sandra Van Dulmen, René M. Vernhout, Lidia R. Arends

**Affiliations:** 1 Department of Internal Medicine, Erasmus MC, University Medical Center Rotterdam, Rotterdam, the Netherlands; 2 Institute of Psychology, Faculty of Social Sciences, Erasmus University Rotterdam, Rotterdam, the Netherlands; 3 Department of General Practice and Elderly Care Medicine, VU University Medical Centre Amsterdam, Amsterdam, the Netherlands; 4 Faculty of Psychology, Open University, Heerlen, the Netherlands; 5 Department of Primary and Community Care, Radboud University Nijmegen Medical Center, Nijmegen, the Netherlands; 6 NIVEL (Netherlands Institute for Health Services Research), Utrecht, the Netherlands; 7 Faculty of Health Science, Buskerud and Vestfold University College, Drammen, Norway; 8 Clinical Trial Center, Erasmus MC, University Medical Center Rotterdam, Rotterdam, the Netherlands; 9 Department of Biostatistics, Erasmus MC, University Medical Center Rotterdam, Rotterdam, the Netherlands; 10 Institute of Pedagogical Sciences, Faculty of Social Sciences, Erasmus University Rotterdam, Rotterdam, the Netherlands; University of Ottawa, CANADA

## Abstract

**Background:**

Patients with medically unexplained physical symptoms (MUPS) are prevalent 25–50% in general and specialist care. Medical specialists and residents often find patients without underlying pathology difficult to deal with, whereas patients sometimes don’t feel understood. We developed an evidence-based communication training, aimed to improve specialists’ interviewing, information-giving and planning skills in MUPS consultations, and tested its effectiveness.

**Methods:**

The intervention group in this multi-center randomized controlled trial received a 14-hour training program to which experiential learning and feedback were essential. Using techniques from Cognitive Behavioral Therapy, they were stimulated to seek interrelating factors (symptoms, cognitions, emotions, behavior, and social environment) that reinforced a patient’s symptoms. They were taught to explain MUPS understandably, reassure patients effectively and avoid unnecessary diagnostic testing. Before and after the intervention training, specialists videotaped a total of six consultations with different MUPS patients. These were evaluated to assess doctors’ MUPS-focused communicating skills using an adapted version of the Four Habit Coding Scheme on five-point Likert scales. Participants evaluated the training by self-report on three-point Likert scales. Doctors in the control group received training after completion of the study.

**Results:**

123 doctors (40% specialists, 60% residents) and 478 MUPS patients from 11 specialties were included; 98 doctors completed the study (80%) and 449 videotaped consultations were assessed. Trained doctors interviewed patients more effectively than untrained ones (*p* < 0.001), summarized information in a more patient-centered way (*p* = 0.001), and better explained MUPS and the role of perpetuating factors (*p* < 0.05). No effects on planning skills were found. On a 3-point scale the training was evaluated with 2.79.

**Conclusion:**

MUPS-focused communication training increases the interviewing and information-giving skills of medical specialists. We recommend that the training is incorporated in postgraduate education for medical specialists and residents who frequently encounter patients with MUPS.

**Trial Registration:**

Dutch Trial Registration NTR2612

## Introduction

Over 50% of newly referred patients to outpatient clinics experience symptoms for which the medical specialist lacks a medical explanation[1]. Medically unexplained physical symptoms (MUPS) are considered to be a working hypothesis based on the (justified) assumption that somatic and/or psychiatric pathology is adequately excluded[2–3]. While most MUPS disappear within a few months, they can endure for more than one year, and become chronic in 20 to 30% of the patients[2, 4].

Medical specialists often find patients whose symptoms have no underlying pathology difficult to handle, and feel incompetent to find agreement with their patients on problem definition[5–7]. The fear of missing a physical disease triggers them to continue medical interventions on MUPS patients, although they achieve low health outcomes and even iatrogenic damage[8–9]. On the other hand, many patients with MUPS do not feel understood, and are offended by messages about the supposed non-somatic origins of their symptoms. They experience a lack of empathy and acceptance for their physical symptoms, and suffer as much as patients with a chronic disease[10–11]. In short, MUPS are a burden to patients as well as to health professionals[12].

Patient-centered communication in MUPS consultations in secondary care has been found to improve patient outcomes and decrease medical consumption[13]. To effectively explore patients’ symptoms and inform patients about the nature of MUPS, medical specialists need specific MUPS knowledge combined with practical communication skills[14–16]. However, general communication training programs in postgraduate medical education lack the content to integrate the do’s and don’ts in MUPS communication. Furthermore, the majority of MUPS research on communication interventions is aimed at primary and mental care. We therefore developed a postgraduate training program for non-psychiatric medical specialists focused on MUPS patients and assessed its effects on their communication skills in a randomized clinical trial[17]. The study question was: Does MUPS-focused communication training facilitate medical specialists to use effective communication in MUPS consultations more often compared to non-trained medical specialists?

## Materials and Methods

### Study design, participants and randomization

We designed a randomized controlled trial to evaluate the effectiveness of a communication skills training for medical specialists to improve MUPS specialist care. A total of thirteen hospitals in the Rotterdam area were invited to cooperate in this study, and six of them were able to respond to our demands and participated. In each hospital one member of the medical staff coordinated the recruitment of doctors; per hospital an electronic flyer for medical specialists and residents with information about the study was sent, and oral presentations were organized if requested.

To participate non-psychiatric medical specialists and residents had to have consultation hours, in which they encountered MUPS patients, and they had to be willing to videotape three MUPS consultations before and after the intervention phase between June 2011 and April 2014.

The medical receptionist briefly informed the patients about the videotaping of the consulting hours. All patients were informed that videotaping was voluntary; they could decide to end it at any time, with their data being deleted immediately upon their request.

The medical specialists and residents were instructed to include new and follow-up patients at the end of a consultation only when ‘physical symptoms were insufficiently explained by pathological findings’.

A team of trained research assistants supported the doctors with recording the MUPS consultations. The unmanned camera was directed at both doctor and patient. After the consultation the research assistant informed the patient about all study related procedures, including further use of the video data and completion of web-based questionnaires. To prevent patient-induced bias during the consultation, more detailed information about the scope of the study was given by the research assistant after the video consultation. A patient information letter was provided, and patients were included in the study only after written informed consent had been obtained. Upon non-participation or withdrawal, all video data were deleted by the research assistant or database administrator (RV). Patient recruitment took place from June 2011-April 2014.

After including up to three MUPS patients, medical specialists and residents were 1:1 allocated by the datacenter on a case-by-case basis to the intervention or control group, using a web-based randomization program. A minimization algorithm was used, ensuring balance within each group and overall balance, with the following stratification factors: medical center and clinical experience (medical specialist versus resident).

Approximately six months after randomization the training for the intervention group was completed, and the research assistants contacted all the specialists and residents to organize the post-measurement videotaping of MUPS consultations. For post-measurements new patients were recruited who had not participated in the pre-measurements. Doctors allocated to the intervention group were trained in MUPS communication skills, whereas doctors allocated to the control group treated patients with care as usual.

### Intervention

The MUPS-focused communication skills training for medical specialists and residents consisted of four sessions with intervals of four to six weeks with a total duration of 14 hours; it has been described elsewhere[17]. The training program consisted of experiential learning, role-play and feedback as crucial elements for learning MUPS-specific communication skills. Using techniques from Cognitive Behavioral Therapy, trainers stimulated the medical specialists to explore patient’s symptoms and search for interrelating factors (symptoms, cognitions, emotions, behavior, and social environment) that reinforced patient’s symptoms. They were taught to inform and reassure patients effectively and offer plausible and understandable explanations for MUPS, reflected in a clear advice and report to the general practitioner. Homework consisted of applying the skills in their consultations, reading literature and watching their own videotaped consultations with MUPS patients prior to the training. An overview of the training program is presented in Table 1.

**Table 1:** MUPS-focused communication training program in specialist care.

***First session*, *4 hr***	**Introduction**
	**Exploring learning goals**
	**Reflection on personal cognitions, emotions and behaviour towards MUPS patients**
	***Practicing skills*:**
	* Exploring biopsychosocial aspects of patient’s symptoms
	* Informing patients about MUPS
	* Explaining vicious circles of perpetuating factors for MUPS
	**Discussion and homework**
***Second session*, *4 hr***	**Exchange of experiences with applying the skills**
	***Practicing skills*:**
	* Reassuring patients effectively
	* Management of expectations prior to additional testing
	* Dealing with complex referrals
	* Report findings in letter to GP
	**Discussion and homework**
***Third session*, *4 hr***	**Peer-review of reply letters to GP about MUPS patients**
	**Treatment of MUPS in Mental Health Care**
	**Presentations in couples of case-material and skills**
	**Discussion and homework**
**Individually**	***Watching of personal videotaped MUPS consultations***
***Fourth session*, *2 hr***	**Exchange of experiences with watching their videotaped consultations**
	**Self-efficacy of their MUPS communication skills**
	**Practicing what is still difficult**
	**Evaluation & SMART intention for keeping skills in shape**

The training was organized in small groups (7 to 12 participants) and provided by one trainer and one assistant trainer experienced in postgraduate education and MUPS skills for medical specialists. In a special workshop the trainers were instructed (AW and AHB) about the training model and the 22 MUPS-focused communication skills, which were divided into (I) interviewing skills to explore biopsychosocial factors, (II) information-giving skills about discussing findings and explaining MUPS and (III) planning skills aimed at follow-up and making appointments. To evaluate the training program, participating doctors were requested to fill out a self-report questionnaire during the last training session on a three point scale (1 = trained MUPS-focused communication skill is not useful, 2 = moderate useful, 3 = very useful).

### Outcome measures on doctors’ communication skills

The application of communication skills was measured by observation of the videotaped consultations. For the assessment we used the validated Four Habits Coding Scheme (FHCS), adjusted in a way that it measured precisely the MUPS communication skills of the training program[18]. We pilot tested this MUPS-focused FHCS in five workshops, in which three trained psychologists (MJ, DA, NK) and the principal researcher (AW) individually assessed each time three different videotaped consultations. The videotapes were discussed. Differences in interpretations were solved by arguments until agreement had been obtained, resulting in a coherent assessment instrument. The application of the communication skills by the medical specialists and residents was rated on a five-point Likert scale. The Likert Scale is a five (or seven) point scale which is used to allow the individual to express how much they agree or disagree with a particular statement. A codebook, S3 Appendix, described every skill on different levels (1 = no or totally inadequate performance of the skill, 2 = imperfect performance, 3 = moderate performance, 4 = good performance, 5 = optimal performance of the skill). Since our Likert scale was symmetric in its categories, with 1 as the lowest score and 5 as the highest score and 3 in the middle, and with the scores 2 and 4 in the middle of “totally inadequate and moderate” and “moderate and optimal”, respectively, it would be likely that the scale would be equidistant and behave as an interval scale measurement. Three trained raters (MJ, DA, NK) blindly scored the videotaped consultations independently, which meant that they did neither have any knowledge about doctor or patient nor about the time the videotape was made (before or after the training period) and the intervention or control status of the doctor. The principal researcher randomly allocated the videotaped consultations to the raters. To obtain adequate inter-rater reliability 50 videotaped consultations were rated by all raters; 120 tapes were rated in couples of two raters to measure inter-rater reliability scores. In 6 rounds the videotaped consultations were rated. After each round, the principal researcher (AW) monitored the quality of the assessments with the raters (MJ, DA, NK), the medical specialist (JS) and the biostatistician (LA) in a workshop.

### Sample size calculation and statistical analysis

To detect a 20% change (from a mean of 2.5 to 3.0, with a standard deviation of 0.8 in both groups) in consultation skills of doctors with a 5% two-sided alpha and a power of 90%, an estimated number of 55 doctors per group were needed (GPower 3.1). Allowing for 10% drop out of doctors we aimed to recruit 60 doctors per group, which meant a total number of 120 doctors.

All analyses were done (LA, JVS, AW) with IBM SPSS Statistics 21.0 i.e. PASW SPSS Statistics 21.0. Nominal variables are presented by frequencies and cross tables. Estimated marginal means and SE’s of the scale scores were calculated for both the intervention group and the control group using linear random effects ANOVA with a random intercept per doctor, thereby taking the clustering of patients per doctor into account. We also fitted this model (SPSS function MIXED with EMMEANS subcommand) to calculate and to compare the differences between pre- and post-measurements for both groups.

### Medical Ethics Review and Approval

The Medical Ethics Research Committee of the Erasmus MC reviewed the study design and officially approved the study and the written informed consent procedures. The Boards of the other five participating hospitals officially agreed to participate in the study, advised by local Medical Ethics Committees. The trial was registered at the Dutch Trial Registration (NTR2612, www.trialregister.nl).

## Results

### Participants

**Fig 1:**
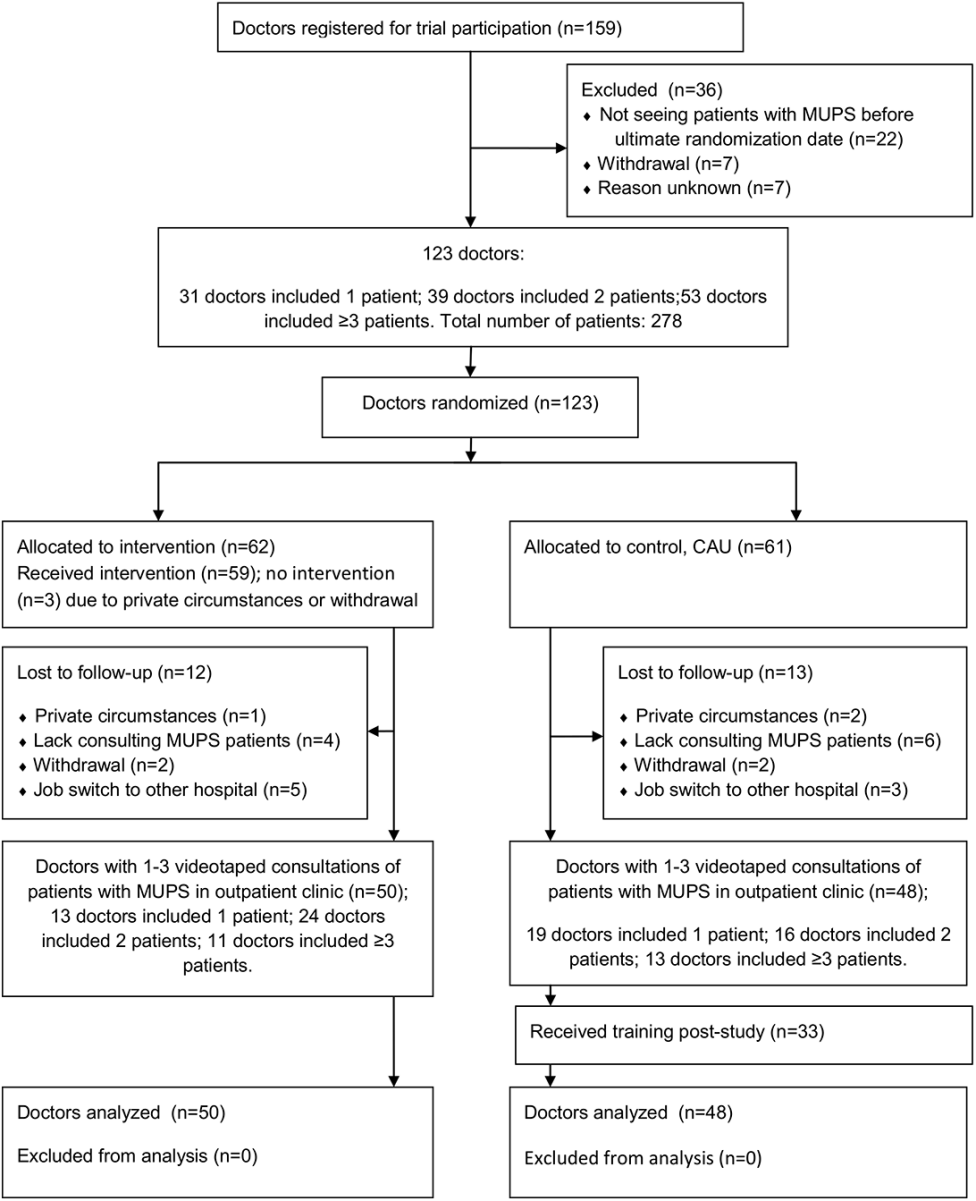
Consort Flow Chart. This figure shows the amount of eligible and included doctors throughout the study accompanied by the amount of videotaped patients per doctor in pre-and post-measurements.

There were 159 doctors (medical specialists and residents) from eleven different specialties eligible for the study between June 2011 and April 2014; 123 of them were able to videotape consultations with MUPS patients at baseline. Sixty-two doctors were allocated to the intervention and 61 to the control group; 98 doctors (50 intervention and 48 control group) completed the study by including one or more patients on videotape (Fig 1). Twenty-five doctors dropped out of the study due to lack of consulting hours with MUPS patients during post-measurements (n = 10), job switch to another hospital (n = 8), withdrawal (n = 4) or private circumstances (n = 3). Doctor baseline demographic characteristics of each group are presented in Table 2. Years of experience has been measured as the number of years between “date of randomization” and “date of start of the education to become a specialist”.

**Table 2:** Doctor characteristics.

*Participating doctors*	Intervention group	Control group
	N = 62	N = 61
*Gender*		
Male	n = 28 (45%	n = 24 (39%)
Female	n = 34 (55%)	n = 37 (61%)
*Resident/Specialist*		
Resident	n = 36 (58%)	n = 38 (62%)
Specialist	n = 26 (42%)	n = 23 (38%)
*Age (SD)*	36.7 (8.9)	36.6 (10.1)
*Years of experience (min*.*–max*.*)*	7.5 (0–31.2)	7.9 (0–34.8)
*Specialism*		
Anesthesiology	n = 2	n = 4
Dermatology	n = 2	n = 0
Gynecology	n = 2	n = 5
Internal Medicine	n = 30	n = 25
ENT	n = 0	n = 4
Lung Diseases	n = 1	n = 1
Gastroenterology	n = 4	n = 7
Neurology	n = 13	n = 9
Rheumatology	n = 6	n = 1
Cardiology	n = 1	n = 0
Rehabilitation Medicine	n = 1	n = 3
*Hospital*		
Albert Schweitzer Hospital Dordrecht	n = 2	n = 4
Diakonessenhuis Utrecht	n = 15	n = 11
Erasmus MC University Medical Center Rotterdam	n = 18	n = 22
Maasstad Hospital Rotterdam	n = 3	n = 2
MC Haaglanden The Hague	n = 13	n = 12
St Antonius Hospital Nieuwegein	n = 11	n = 10

### Videotaped consultations with MUPS patients

A total of 478 MUPS patients participated in the study between November 2011 and April 2014: 278 at baseline and 200 at follow-up. Most patients were female (63%), had an average age of 46 years (SD = 16), had an education on primary (4%), secondary (60%), tertiary level (27%) or other (9%) and visited the outpatient clinics for Internal Medicine (n = 193) or Neurology (n = 94). The included 478 patients provided 520 videotaped consultations: in 42 cases the doctor videotaped two consultations of the same patient within pre- or post-measurements. To maintain independent statistical analysis only the first one of these two consultations was used. A total of 29 videotaped consultations were unsuitable for analysis due to technical imperfections (n = 20), exclusion because patients’ symptoms were explained by a somatic disease (n = 5) or patients’ withdrawal (n = 4). The 449 videotaped consultations were assessed by three psychologists. They rated each video on all 22 items. To check the reliability of the observers during the study, we randomly selected some videos that were-randomly- assigned to two (n = 120 videos) or all three (n = 50 videos) raters. Per videotaped consultation we calculated the ICC across the two or three raters over all 22 items. The mean ICC across all videos was 0.78. Table 3 shows patient characteristics.

**Table 3:** Patient characteristics of assessed videotaped consultations.

*Participating patients*	Intervention group	Control group
	N = 229	N = 220
*Gender*		
Male	N = 76 (33%)	N = 90 (41%)
Female	N = 153 (67%)	N = 130 (59%)
*Age (SD)*	45.9 (16.2)	46.0 (16.2)
*Specialism*		
Anesthesiology	n = 8	n = 16
Dermatology	n = 3	n = 0
Gynecology	n = 5	n = 16
Internal Medicine	n = 106	n = 87
ENT	n = 0	n = 15
Lung Diseases	n = 4	n = 5
Gastroenterology	n = 13	n = 31
Neurology	n = 57	n = 37
Rheumatology	n = 27	n = 0
Cardiology	n = 2	n = 2
Rehabilitation Medicine	n = 6	n = 13

Trained medical specialists and residents showed a bigger increase in exploring patients’ cognitions (*p* < 0.001) and the impact of the symptoms on patients’ behaviors (*p* = 0.001), social environment (*p* < 0.001) and emotions (*p* < 0.001) than the untrained medical specialists and residents. Trained medical specialists and residents also summarized information in a more patient-centered way (*p* = 0.001) and told the patient more frequently about interrelating factors and MUPS (*p* = 0.017) than the untrained specialists and residents. No effects were found on the skills for making plans and follow-up appointments. The skills of exploring physical symptoms, acknowledging the reality of patient’s symptoms, explaining doctor’s perspective concerning symptoms and treatment options, allowing time for information to be absorbed, summarizing appointments and displaying nonverbal empathy were already reasonably apparent in both groups before randomization. Table 4 shows the effects on doctors’ MUPS communication skills.

**Table 4:** Effects on doctors’ communication skills. Estimated marginal means on scale range 1–5; 1 = min., 5 = max. score. Intervention group = IG, Control group = CG, pre = pre-measurement, post = post-measurement. P-value contrast: ‘IG post-training minus IG pre-training’ compared to ‘CG post-training minus CG pre-training’

MUPS communication skills	IG_pre	IG_post	CG_pre	CG_post
	N = 137	N = 92	N = 125	N = 95
	Mean (SE)	Mean (SE)	Mean (SE)	Mean (SE)
*I Interviewing skills focused on exploring biopsychosocial factors*				
I.1 Interested in the patient’s understanding of the problem***	2.3 (0.15)	3.6 (0.17)	2.4 (0.15)	2.6 (0.17)
I.2 Shows interest in impact of symptoms on patient’s activities/behavior**	2.5 (0.16)	3.5 (0.19)	2.5 (0.16)	2.6 (0.19)
I.3 Shows interest in impact of symptoms on patient’s social environment***	1.5 (0.13)	2.7 (0.15)	1.6 (0.13)	1.7 (0.15)
I.4 Encourages expression of emotions related to symptoms***	2.2 (0.15)	3.0 (0.17)	2.2 (0.15)	2.0 (0.17)
I.5 Explores physical symptoms	3.5 (0.17)	3.7 (0.20)	3.7 (0.17)	3.5 (0.20)
I.6 Acknowledges the reality of patient’s symptoms	3.5 (0.11)	3.8 (0.13)	3.6 (0.12)	3.5 (0.13)
***TOTAL*** ***	***15*.*5 (0*.*60)***	***20*.*2 (0*.*69)***	***16*.*0 (0*.*62)***	***16*.*01(0*.*69)***
*II Information-giving skills about findings and explaining MUPS*				
II.07 Summarizes information according all SCEBS items using patient's perspective**	1.1 (0.06)	1.5 (0.07)	1.2 (0.06)	1.1 (0.07)
II.08 Frames information in positive language	2.8 (0.15)	3.2 (0.18)	2.8 (0.15)	2.8 (0.18)
II.09 Explains symptoms are not caused by disease	2.1 (0.15)	2.8 (0.18)	2.2 (0.16)	2.4 (0.18)
II.10 Explains perpetuating factors*	1.8 (0.14)	2.7 (0.16)	1.8 (0.14)	2.0 (0.16)
II.11 Uses drawings in the explanation of MUPS	1.1 (0.06)	1.3 (0.07)	1.0 (0.06)	1.1 (0.07)
II.12 Acknowledges perspectives of patient concerning symptoms and treatment options	2.8 0.14)	3.1 (0.17)	2.6 (0.15)	2.5 (0.17)
II.13 Explains perspectives of doctor concerning symptoms and treatment options	4.2 (0.12)	4.2 (0.14)	4.2 (0.12)	4.1 (0.14)
II.14 Connect perspectives of doctor AND patient	2.5 (0.15)	2.9 (0.17)	2.3 (0.15)	2.4 (0.17)
II.15 Allows time for information to be absorbed	3.7 (0.12)	3.6 (0.14)	3.8 (0.12)	3.7 (0.14)
***TOTAL*** *	***22*.*1 (0*.*70)***	***25*.*4 (0*.*82)***	***21*.*7 (0*.*72)***	***22*.*2 (0*.*82)***
*III Planning skills concerning follow-up and appointments*				
III.16 Explains rationale and possible outcomes of test results prior to testing	2.6 (0.16)	2.8 (0.19)	2.6 (0.17)	2.5 (0.19)
III.17 Effectively tests for comprehension	3.0 (0.13)	3.1 (0.15)	3.0 (0.13)	2.8 (0.15)
III.18 Encourages involvement in decision-making	2.2 (0.13)	2.1 (0.15)	2.1 (0.13)	2.0 (0.15)
III.19 Explores acceptability of treatment and/or follow-up plan	2.5 (0.13)	2.2 (0.16)	2.4 (0.13)	2.4 (0.15)
III.20 Explores barriers to implementation of treatment and/or follow-up plan	1.6 (0.10)	1.6 (0.13)	1.7 (0.11)	1.6 (0.12)
III.21 Summarizes plans for follow-up	4.0 (0.13)	4.0 (0.15)	4.2 (0.13)	4.0 (0.15)
III.22 Displays effective nonverbal empathy in the whole consultation	4.2 (0.11)	4.3 (0.13)	4.0 (0.11)	3.9 (0.13)
***TOTAL***	***20*.*1 (0*.*54)***	***19*.*9 (0*.*64)***	***19*.*9 (0*.*56)***	***19*.*4 (0*.*63)***

*** = *p* < 0.001

** = *p* < 0.01

* = *p* < 0.05

### Evaluation of the training program

Medical specialists and residents appreciated the training program as very useful for daily practice. The intervention group lost 3 doctors before the training started. A total of 92 doctors received the MUPS communication training (doctors of the control group were offered the training after they finished all measurements, and 33 of them obtained the training). They evaluated the usefulness of the training, concerning exercises, skills, literature, duration and feedback, with 2.79 [CI, 2.75 to 2.83] on a three point Likert scale (with 1 as minimum and 3 as maximum score).

Despite the value of the MUPS communication skills for daily practices, medical specialists and residents experienced consultations with MUPS patients from different ethnic background as extremely difficult.

## Discussion

### Main findings

The main aim of the study was to evaluate the effectiveness of our MUPS-focused training for medical specialists on doctors’ communication skills, which to our knowledge is a novelty in secondary care. The results clearly indicate that medical specialists who had taken the training had better interviewing and information-giving communication skills in MUPS consultations than those who had not. Participants rated the training as very useful.

### Comparison with literature

Our findings are in line with research in general care. Aiarzaguena et al. showed that GPs benefit from a MUPS-focused communication training program[19–20]. GPs valued two key elements: the structure, which facilitated a more comfortable relationship with MUPS patients; and the options of transferring the skills to a broader spectrum of patients with psychosocial problems. Rosendal et al. developed a brief MUPS-focused training program for GPs, which changed GPs' attitude towards patients with somatoform disorders[21]. Rief et al. designed a one-day workshop for GPs on managing MUPS patients, in which the topics included how to communicate with MUPS patients, and when to start and stop medical examinations and treatment options. GPs valued this workshop as highly relevant to their daily practice[22]. By stressing that postgraduate education in MUPS-focused knowledge and communication skills is both relevant and necessary in general care, these studies reinforce the importance of our results for specialist care.

### Strengths and limitations

A strength of our study is the fact that our engagement of 123 doctors from six different hospitals and eleven specialties in the study enabled us to assess the effectiveness of the training in different medical settings. To reach this number of participants, we decided to switch from a single to a multi-center study design, and extended the inclusion period by a full year.

In our search for a valid instrument to assess specialists’ communication skills we chose the Four Habits Coding Scheme (FHCS) and integrated the defined 22 MUPS communication skills within this FHCS. By measuring precisely the skills that were subject of the training program, this enriched MUPS-FHCS contributed to the strength of this study. Fossli Jensen et al. assessed the effectiveness of a 20-hour training model for hospital doctors also with the Four Habits Coding Scheme, and found comparable levels of improvement of clinical communication doctors’ skills [23]. Our training model is even shorter (14 hr) and efficient especially on exploring, which we consider to be a strength.

A further strength of the study is that our outcomes at doctors’ level were rated at the highest level of performance according to Miller. This four-level scale discerns *knows* (level 1), *knows how* (2), *shows* (3) and *does* (4). The *does* level refers to measurement of clinical performance in real practice[24].

This study also has weaknesses. One limitation concerns the average number of videotaped consultations per doctor, which was lower than we had expected. The three MUPS patients per measurement per doctor were not always achieved. Sometimes patients refused to be videotaped or didn’t show up at consulting hours. However, 449 MUPS consultations for analysis appeared to be enough to prove the effectiveness of the training.

In their consultations with patients from different ethnic backgrounds, doctors felt hampered by the lack of three prerequisites: time, professional interpreters and knowledge of cultural diversity. The doctors spoke Dutch language in the videotaped consultations. Most of today’s patients in big cities, such as Rotterdam, are culturally rooted in other countries. It may therefore be a weakness that the cultural sensitivity of the training program was not more developed. The fact that most MUPS patients are female may explain why doctors used these MUPS skills more in consultations with female patients than in those with male patients.

### Conclusion

Our MUPS-focused communication training significantly increases the interviewing and information-giving skills of medical specialists and residents. We conclude that we have developed a feasible and effective training program that enables medical specialists and residents to improve their consultations with MUPS patients. We therefore recommend that the training model is incorporated in postgraduate education for medical specialists and residents, who often encounter such patients.

### Implications for future research

Due to the presentation of their own case material during the training, medical specialists achieved greater awareness of MUPS in outpatient clinics. In some cases they noted that use of care by patients with persistent MUPS was extremely high: some patients had consulted the Emergency Department (ED) and other specialties up to 20 times in the previous three months. Future research on the prevalence and follow up of MUPS patients in the ED will help to identify patients’ needs, and indicate how MUPS care should improve in specialist care.

Future studies and training programs should reconsider the cultural and gender sensitivity of the training model, and adjust it where necessary.

## Supporting Information

S1 Appendix CONSORT 2010 Checklist. (DOC)

S2 Appendix Study protocol, English summary. (DOC)

S3 Appendix Code Book for assessing MUPS videotaped consultations. (DOC)

S4 Appendix Original Study protocol, partly in Dutch language. (PDF)
