# Supplementary material for: Training Medical Specialists to Communicate Better with Patients with Medically Unexplained Physical Symptoms (MUPS). A Randomized, Controlled Trial
Source: PLoS One. 2015 Sep 18;10(9):e0138342. doi: 10.1371/journal.pone.0138342 (PMC4575077; doi:10.1371/journal.pone.0138342)
Supplement: S2 Appendix — (DOC) [file pone.0138342.s002.doc]

# This supplement belongs to manuscript ‘Training medical specialists to communicate better with patients with medically unexplained physical symptoms (MUPS). A randomized, controlled trial.

**This supplement contains the following items:**

1. **Original Research Protocol including Statistical Analysis**
2. **Summary of changes**

**1. Original Research Protocol including Statistical Analysis**

Project title **Effectiveness and efficiency of a consultation skills training programme for medical specialists focused on patients with medically unexplained physical symptoms (MUPS).**

Date of grant-application **02-02-2010**

Date of grant-acceptance **01-11-2010**

Protocol Approval **10-01-2011**

Start research **01-04-2011**

Planned duration **24 months**

**Summary project information**

*Objective*:

To improve medical specialist care for patients with medically unexplained physical symptoms (MUPS) by developing and researching the effectiveness and efficiency of a consultation skills training programme for medical specialists focused on MUPS-patients.

*Study questions*:

1.What is the effect of training medical specialists in MUPS-focused consultation skills on illness worries, course of symptoms, and daily functioning of MUPS-patients in comparison with non-trained specialists?

2.Do trained doctors use more effective communication compared to non-trained ones?

3.Is the training course cost-effective?

*Design*:

RCT with individual randomization and intervention on doctor-level and effect measurement on patient- and doctor-level.

*Study population*:

1. Medical specialists and trainees of Erasmus MC.

2. Out-patients with MUPS, diagnosed by the participating specialist.

*Intervention*:

MUPS-focused consultation skills training course for medical specialists.

*Outcome measures:*

Q 1: On patient-level with questionnaires we measure: illness worries with Whitely Index; current symptom severity with VAS; distress, depression, anxiety and somatisation with 4-DSQ; daily functioning of patients with SF-36.

Q 2: On doctor-level we measure the application of consultation skills by observation of video-taped consultations and facts from patient files.

Q 3: An economic evaluation will be performed from societal perspective. Costs of training programme, health care utilization, personal expenses, production loss will be measured. The research team registers costs of intervention; we measure the other volumina by patient questionnaires.

*Sample size calculation*:

To detect a 20% improve in consultation skills of doctors with a 5% two-sided alpha and a 10% beta, an estimated number of 55 doctors per group will be needed. Allowing for 10% drop-out of doctors we aim to recruit 60 doctors per group. On patient level a sufficient study power will be reached. On the SF-36 outcome measure a minimal difference of 10% is considered relevant and can be detected with 110 patients per group. We plan 129 patients per group per measurement to be available for analysis.

*Time-schedule*:

Month 1-2 preparation, 3-20 data collection and intervention, 21-24 analysis and report.

*Cooperating organizations:*

Erasmus MC, University Medical Centre Rotterdam, Department of Internal Medicine

Erasmus University Rotterdam, Faculty of Social Sciences, Institute for Psychology

Netherlands Institute for Health Services Research (NIVEL)

VU University Medical Centre Amsterdam, Department of General Practice and Elderly Care Medicine

**Content**

**Problem definition**

Having physical symptoms is quite normal: in population surveys 85-95% of respondents report at least one symptom during the preceding week. The decision to seek medical help is determined more by cognitions about cause, prognosis or need for medical intervention, than by severity of the symptom. In 30-50% of the physical symptoms presented in general practice the symptoms remain medically unexplained (MUPS). In hospital practice this figure is even higher: 40-60% of the presented symptoms remain medically unexplained. Although most of these symptoms disappear within a few weeks or months, still 20-30% of MUPS persist for more than a year. Patients with persistent MUPS often have reduced subjective health with impaired physical, mental or social functioning, increased prevalence of co-morbid depressive or anxiety disorders and increased sick-leave (De Waal 2004). MUPS are more prevalent among women and associated with older age, low education, employment disability or unemployment (Verhaak 2006).

Many patients with persisting MUPS have feelings of not being taken seriously by their doctors, while their doctors often feel unable to find common ground and to get agreement on a common problem definition (Salmon 2007). Patients are easily offended by incongruent messages about a supposed non-somatic origin of the symptoms, experiencing lack of empathy and acceptance for the physical symptoms. Cultural and language barriers may increase the communication problems. These doctor-patient communication problems can hamper a proper exploration of problems and an adequate treatment for persistent MUPS.

Several studies of patients' perspective on consultation showed the importance of patients seeking legitimacy for their problems (Zavestoski 2004, Peters 1998). Patients want to feel that their doctor accepts that the symptoms are real and they want doctors' attention for that. Whereas patients seek legitimacy for their physical symptoms, many doctors regard these symptoms as not warranting their help or they attribute the symptoms to psychological problems. Whereas patients want engagement, doctors have described feeling that engagement is pointless. Doctors fluctuate in their willingness to engage with patients' emotional cues depending on mood or the pressure of work (Cocksedge 2005).

Many patients with MUPS demand for doctors engagement will not be satisfied in that need. Dissatisfaction and pressure on the doctor-patient communication are often the results. Qualitative analyses have shown how doctors' explanations can create common ground that allows patients and doctors to address both psychosocial and physical problems and to avoid somatic interventions (Dowrick 2004, Salmon 1999). In a randomized, non-blinded study patients who received a firm diagnosis for their symptoms were more likely to improve than those who received no explanation (Thomas 1987). Simple explanation by the doctor or nurse has improved reassurance by normal tests (Petrie 2007).

Teaching about explanation in medical education is limited. Explanation as a skill is underestimated in the curriculum. In order to improve the care for MUPS patients the skills of explanation of MUPS and reassurance has to be developed not only by GPs but also by medical specialists.

**Relevance**

Of patients presenting physical symptoms in outpatient clinics, such as gynecology, neurology or rheumatology, at least 40% of patients have no physical disease (Nimnuan e.a., 2001; Maiden, 2003; Reid, 2001).

These symptoms burden patients as well as health services due to large quantities of physical intervention, even surgery, which is often ineffective (Stanley, 2002; Barsky, 2005).

The health outcome of patients with MUPS can be influenced positively by patient centered communication, effective reassurance, reliable patient information and a positive explanation (Dowrick e.a., 2004; Fassaert e.a., 2008). The problem is that doctors primarily use a medical approach in their consultations with MUPS patients and reinforce unconsciously the attention of the patients towards the somatic nature of their ailments (Kappen & Van Dulmen, 2008).

To identify the basis of the communication problems that characterize consultations with MUPS patients a literature review on this subject showed that contest between patients' authority, resting on their knowledge of their symptoms and practitioners' authority, based on the normal findings of tests and investigations often is very much involved (Salmon, 2007). The outcome of consultations can be influenced by the strategies that patient and doctor use to press their authority, rather than on clinical need. Finding sensible, common ground in the explanation of the symptoms, shared by doctor and patient, is therefore very much indicated as a preferred strategy. The practitioner needs to fashion explanation that is acceptable to both parties from

available medical and lay material. That means clinical education programmes should include curricula in symptom explanation focused on MUPS- patients.

The intervention under study is an effort to provide in such an education programme for medical specialist and trainees. The curriculum for medical specialists lacks the subject of consultation skills training for MUPS patients. The pilot of this training programme for neurologists in the Erasmus MC, described under 'clinical study', created a unique opportunity for the Central Education Committee to improve the quality of the interdisciplinary medical specialist care for MUPS-patients.

The proposed study meets the criteria of the VEMI programme because the patient group with MUPS is at risk to become a frequent and long term user of medical health services. In case of ailments like CFS, IBS and fibromyalgia the diagnostic process usually takes a long time with extra unnecessary tests, which is costly for the society and a personal burden for the patient.

Because medical interventions are not always available for patients with MUPS communication is even more important to maintain an adequate doctor-patient relationship and contribute to an effective medical care for MUPS patients. The new national multidisciplinary guideline on MUPS and somatoform disorders (MDR Trimbos-institute, CBO, 2009) advises a dual policy (exploration of both the somatic aspects and psychological aspects such as the cognitions, emotions, illness worries and impact of symptoms for daily functioning of the patient), effective reassurance, positive explanation, adequate registration and report to the GP.

Effective consultation focused on MUPS-patients prevents the development of somatic disorders, improves the quality of life of MUPS-patients and the realization of cost reduction due to the prevention of useless medical specialist interventions.

The intervention in the proposed study contains a consultation skills training programme for medical specialists according to the above mentioned national multidisciplinary evidence based guideline on MUPS and somatoform disorders.

**Objective**

The aim of the study is: To improve specialist care for patients with MUPS by development and research of the effectiveness and efficiency of a consultation skills training programme for medical specialists focused on patients with MUPS.

The primary study question is:

1. What is the effect of training medical specialists in MUPS-focused consultation skills on illness worries, course of symptoms and daily functioning of MUPS-patients in comparison with non-trained specialists?

Secondary study questions are:

2. Do trained medical specialists use more effective communication compared to non-trained ones?

3. Is the training course cost-effective?

**Strategy**

CLINICAL STUDY

Preliminary studies by researchers:

Blankenstein performed a cluster-randomized RCT with 162 somatising patients on effectiveness of cognitive-behavioral techniques applied by trained GP to patients with MUPS. In a 20-hour GP training programme, intervention GPs learned to apply reattribution combined with a cognitive-behavioral approach to persistent illness worries. This appeared to be a feasible intervention that could be applied during normal surgery hours. In the RCT the intervention was compared to usual GP care.

From baseline to two-year follow-up, subjective health increased in the intervention group from 36/100 to 74/100, and from 36/100 to 39/100 in the control group (p<.001). Total number of health care visits during the preceding 6 months diminished in the intervention group from 10 to 5, and remained stable in the control group: from 12 to 13 (p.017). Sick leave in weeks during the preceding 6 months decreased in the intervention group from 5 to zero, and remained stable in the control group: from 4 to 4 (p<.001). Use of medication did not change in either of the groups. (Blankenstein, 2002). Blankenstein has also contributed to a community survey on impact of physical symptoms on perceived health with 2447 responders (response 53%). Fatigue was the most commonly reported symptom with a prevalence of 57%, followed by headache (40%) and low back pain (39%). More than half of responders reported three symptoms or more. Increasing number of symptoms is strongly associated with poorer physical, emotional and social functioning. (Van der Windt 2008).

Blankenstein is currently performing a Cochrane systematic review on psychiatric consultation letters for MUPS. (Hoedeman, Blankenstein 2007). Blankenstein performed a study on determinants of successful patient recruitment in research [van der Wouden, Blankenstein et al. 2007]

Apart from this the project advisor on this proposed study found that consultations for medically unexplained physical symptoms (MUPS) constitute a large part of the workload of a broad scale of medical specialists, i.e. internists, gastroenterologists, gynecologists, pediatricians, etc. As medical interventions are not always available, specific communication strategies might help physicians to handle these problems adequately. Although such a strategy seems quite logical, recent research of the NIVEL communication group examining ninety-seven videotaped medical visits from patients presenting MUPS shows that physicians primarily use a medical approach in their communication (Kappen & Van Dulmen,2008). Such an approach might incorrectly confirm the somatic nature of the patient’s condition. As results also show that most patients present their concerns only in an implicit way, physicians may need to be taught to recognize and explore patients’ concerns more actively. Findings from another NIVEL study indicate that such an approach might indeed be successful with MUPS-patients. In this study, the use of positive communication strategies during 524 videotaped consultations with patients with minor ailments was analyzed and related to the visit outcome in terms of medication adherence, consultation frequency, functional health status and state anxiety. Results show that, to some extent, it seems helpful when GPs are at the same time clear and optimistic about the nature and course of minor ailments. Although communication strategies might to some extent contribute to the management of minor ailments, the results of this observational study also indicate that it is important for a physician to pay attention to the mood of the patient who enters the consulting room (Fassaert et al, 2008). Previous research with 120 patients with IBS visiting an internist indicates that for this purpose a minimal psychological intervention provided by a physician can already be effective in ameliorating MUPS symptoms (Van Dulmen et al, 1994; 1996; 1997). These results will be included in the training which will be offered to the medical specialists in the proposed study.

Weiland performed a pilot of the intervention ' consultation skills training programme for medical specialists focused on MUPS-patients' in cooperation with the Department of Neurology Erasmus MC Rotterdam (2007, 2008). In two different groups a total of 22 neurologists (5 staff members and 17 medical specialist trainees) participated in this training with an attendance rate of 90%.

In 4 training sessions with an interval of 4 to 6 weeks the neurologists and trainees learnt consultation skills focused on MUPS-patients, which they practiced during consulting hours in between the sessions. The training model was based on learning-by-doing and had the character of workshops, in which step by step new skills were introduced, practiced and supervised. In small groups of three participants role-plays were the educational instrument (doctor-patient-observer). The use of individual case-material was enhanced.

In session 1 the basics of MUPS focused consultation skills were taught:

- Exploring of patients complaints following the SCEBS-model (SCEBS: Somatic symptoms, Cognitions, Emotions, Behavior and Social environment).

- Informing the patient in positive vocabulary, with referral to the patients cognitions/emotions and with clear explanations such as “the lungs are clean and I hear a healthy sound of your breath”. Special attention was given to vicious circles and plausible explanations.

- Planning and making appointments with patients in a SMART way: little steps ahead which can be accomplished successfully.

In session 2 gathering more information of the patient by registration of its own complaints and searching for new connections together with the patient was practiced:

- Purpose of the registration

- Motivating patients

- Giving clear instructions

- Design of the registration

- Planning and discussing with the patient.

- Reattribution: maximization of patients reception to a new understanding of his complaints and acceptance of other explanations of the symptoms than ‘it must be a disease’.

In session 3 skills for patients with persistent illness worries were introduced and report to the referring doctor was discussed:

- The art of reassuring patients

- Questioning patients about their dramatic thoughts

- Discussing the impact of these scenarios

- Putting these thoughts to the test (reality-check)

- Evaluation: change in patient perception of complaints

- Managing expectations about possible test results

- Addressing health anxiety

- Dealing with referral letters

- Report to GP

In session 4 the participants presented their experiences with the new knowledge and skills:

- Review of treatment of a patient with the accomplished skills in couples

- Discussion and feedback

- Practicing skills when necessary

The pilot was evaluated by a questionnaire (N=19). Results of the evaluation in terms of useful (not, medium or very useful) show:

* Recommendation of the training model (practicing-step-by-step and learning-by-doing) due to supervision of trainers and feedback of participants.

* Exploring 'SCEBS' as the most useful experienced skill because its impact on control for the doctor in the communication with (all kind of) patients (by 18 participants).

* Practicing of reassuring patients effectively was evaluated as very useful (by 16 participants).

* Informing patients about possible interrelating factors reinforcing their complaints was as skill equally evaluated with the management of expectations (14 participants said very useful).

* The participants evaluated the skills ‘planning SMART activities with patient’ and ‘reattribution’ as medium useful (by 11 participants) due to logistical problems in the follow-up of out-patients.

* Adjusting the reader for medical specialists was evaluated as an improvement for the course.

* Guided intervision as a follow-up of the training was mentioned as a need for permanent education on this subject.

Results were presented to the Head of Department of Neurology and the Central Education Committee Erasmus MC. The pilot-training was discussed in the research team and adjustments for improvement of the intervention have been made for this proposed study.

DESIGN:

RCT with randomization and intervention on doctor-level and effect measurement on patient- and doctor-level.

Setting: Out-patient clinic of the Erasmus MC.

Study population: medical specialists and trainees within Erasmus MC and their out-patients with MUPS.

INTERVENTION:

Training consultation skills for medical specialists focused on patients with MUPS.

The specialists receive 4 training sessions, with an interval of 4 to 6 weeks, in groups of 12 persons with 2 experienced trainers (trainers receive special training and supervision); 5 intervention- and 5 control groups will be composed. After individual randomization of doctors 10 training groups will be composed in a way that doctors of different medical specialism’s are mixed together in a group, in order to stimulate the broader scope, which is necessary for patients with MUPS.

The medical specialists will be taught:

1- to notice and respond to the concerns of the patient

2- to register these concerns in the (electronic) patient record

3- to address the reasons for referral

4- to reassure the patient

5- to give a positive explanation, in which the doctor is emphasizing that and how symptoms frequently occur without medical

diagnosis or disease.

6- to speak clearly and motivational with the patient

7- to write a letter to the patient's GP in which the explanation given to the patient is resumed, and recommendations on policy and lifestyle are reported.

For the items 1, 4, 5 and 6 the evidence based course of the Dutch College of General Practitioners ‘GP-policy for medically unexplained physical symptoms’ can be used with slight adjustments, for items 2, 3 and 7 new education will be developed.

Doctors in the control group receive the training consultation skills afterwards, when the RCT is finished.

Recruitment of doctors:

Medical specialists and trainees of the participating medical specialism’s are included in the research on the premises that (1) they are prepared to participate in the training and the measurements before and after the training; (2) they agree that their MUPS-patients will be asked to participate in the research; (3) they perform consultations with out-patients during the research period.

Recruitment of patients:

Doctors are video-taping consultations with an unmanned camera during the out-patient clinic with informed consent of the patient. After every consultation the doctor will determine whether the physical symptoms of the patient can be insufficiently explained by a medical disease (= MUPS, most likely 30-50%). These MUPS-patients will be asked to participate in the research (pre-training group). After training again doctors will video-tape consultations and patients again will be asked to participate in the research (post-training group). The patients in the post-training group are not the same nor overlapping with patients in the pre-training group.

A total number of 720 MUPS-patients will be video-taped and, when informed consent is given by these patients, analyzed.

With a response rate of 80% the questionnaires of 576 patients will be analyzed.

To avoid failing patient recruitment by busy doctors, students will be recruited to organize the recording of video-tapes, and to ask informed consent from eligible patients.

The inclusion period consists of 5 rounds: during 5 months every month 24 specialists and trainees with MUPS-patients will be included.

Randomization: at the end of every inclusion round, after recording of video consults and inclusion of pre-measurement patients, the 24 doctors of that round will be individually randomized and stratified for medical discipline and medical specialists versus vocational trainees. Assigning by lot, by an independent person, 12 doctors with their MUPS-patients will be allocated to

an intervention- and 12 to a control group.

FEASIBILITY OF DOCTOR RECRUITMENT:

Based on the outcome of an inquiry by questionnaire in the Erasmus MC the feasibility of doctor recruitment is sufficient. Nine medical specialism’s are willing to schedule the training 'consultation skills for medical specialists focused on MUPS patients' for their staff and trainees (total number of available participants is 134 trainees and 18 medical specialists; for the trainees the training will be part of the interdisciplinary curriculum). Participation will be stimulated actively by prof. dr. J.L.C.M. van Saase, head of the Internal Medicine education programme and Chair of the Central Education Committee Erasmus MC.

Feasibility of the time-schedule is also sufficient: intervention already is almost developed, data collection can start quickly and being accomplished within 15 months. Analysis and report take place while control groups receive their training. Sufficient supporting research assistants are a necessity to perform the project in 2 years in the Erasmus MC.

OUTCOME PARAMETERS:

Research question 1. On patient-level we will measure: illness worries of patients about their complaints with the Whitely Index; symptom severity with VAS scale(s) for the current symptom(s; distress, anxiety, depressive symptoms and somatisation with the 4-DSQ [Terluin]; impact of symptoms on daily functioning with the SF-36.

Research question 2. On doctor-level we will measure the practicing of consultation skills in real-patient consultations with MUPS patients: trained observers will score items 1, 3, 4, 5 and 6 by observation of video-taped consultations. A standardized observation scoring list will be developed, based on the Roter Interaction Assessment System (RIAS), containing only those aspects relevant for measuring item 1, 3, 4 and 5; items 2 and 7 will be extracted from the patient records. To enhance the reliability of the consultation skills assessment, before the training 3 consultations with MUPS patients and after the training another 3 consultations will be video-taped and assessed for every doctor. Fifty videotaped consultations will be double observed and scored to assess the interrater reliability.

Research question 3. An economic evaluation will be performed from societal perspective. As costs will be involved the direct costs of the intervention (training course + time-investment of medical specialists), costs of healthcare (number of attending to GP, medical specialists, Mental Health Care, paramedical care, alternative healers; hospital admission days; hours homecare; prescribed medicines), costs for patient and family (transportation costs, hours family care, self medication), and indirect costs due to loss of production (absenteeism and presenteeism). The research team registers the intervention costs, the other volumina will be measured with patient questionnaires.

Measurement timings and - procedures:

In the month after inclusion of the doctors pre-measurement consultations will be videotaped and the patients with MUPS in the pre-training group will be included. These patients will be asked to answer the questionnaire directly after the consultation and after 3 and 6 months. In the meantime the doctors in the intervention group receive the training.

Six months after the inclusion of the doctor the post-training consultations will be videotaped and the patients with MUPS in the post-training group will be included. These patients also will be asked to answer the questionnaire immediately after the consultation and after 3 and 6 months. For logistic reasons students will be involved during the process of data collection.

**Statistical analysis**

Research question 1. Differences between intervention- and control patients (clustered within doctors) will be measured with the WI, VAS, 4-DSQ and SF-36 at 3 and 6 months in the pre training- and post training groups; differences will be addressed at baseline with multilevel analysis on doctor- and patient-level (SAS Proc Mixed).

Research question 2. Separate scores on the 7 items each as well as a sum score on consultation skills will be analyzed at doctor-level. Therefore item- and total scores of 3 consultations (patients) in the pretraining group and 3 consultations (patients) in the post training group of the same doctor will be compared. Differences in the post training scores between intervention- and control doctors will be adjusted for their pretraining scores, and will be analyzed with the General Linear Mixed Model (SAS Proc Mixed). Contrast analyses will be planned for the demographic characteristics of the patients, whether it is a first versus a follow-up consultation of the patient, and (senior) specialists versus trainees.

Research question 3. If available, costs will be calculated using standard prices (Oostenbrink list). Care by family members will be calculated by using shadow prices. Total costs per patient will be estimated on the basis of direct as well as indirect costs. Analysis will include bootstrapping techniques. Results will be presented as incremental cost effectiveness ratios, of which reliability will be presented in cost effectiveness diagrams.

SAMPLE SIZE CALCULATION:

To detect a 20% improvement in consultation skills of doctors with a 5% two-sided alpha and a 10% beta, an estimated number of 55 doctors per group will be needed. Allowing for 10% drop-out of doctors we aim to recruit 60 doctors per group. Because we have three pretraining patients and three post raining patients per doctor, on patient level a sufficient study power will be reached easily. On the SF-36 outcome measure a minimal difference of 10% is generally considered as clinically relevant. This difference can be detected with 110 patients per group. We aim at 129 patients per group, both for pre- and for post-measurement available for analysis.

ECONOMICAL EVALUATION

-General considerations

To judge the training in terms of its cost-effectiveness, where effects on 'quality of life' are expressed in terms of money, we will perform cost-effectiveness analysis. Besides, we expect reduced costs for medical treatment in the intervention group when we compare the post training group to the pretraining group. To see whether the benefits of reducing the direct and indirect costs outweigh the costs of the training, both in terms of money, we will also perform a cost-benefit analysis.

-Cost analysis

We will calculate the costs of the burden of disease of MUPS for the patients in this project with a questionnaire on direct and indirect costs associated with MUPS measured over last month. Direct costs of the intervention are e.g. the costs of the trainings course, the time-investment of medical specialists and of the trainer. Other direct costs concern the costs of resources for medical treatment including prescribed medicines and alternative healers.

Finally we will measure costs for patient and family (transportation costs, hours family care, self medication), and indirect costs due to loss of production (absenteeism and presenteeism). The research team registers the intervention costs, the other volumina will be measured with patient questionnaires per month.

-Patient outcome analysis

To measure the effects of the training we will use following instruments: the WI, VAS, 4-DSQ and SF-36 at baseline, after 3 months and after 6 months in the pretraining group (intervention- as well as control group) as well as the post training group (intervention- as well as control group).

SYSTEMATIC REVIEW:

In the intervention of the proposed study, a consultation skills training programme for medical specialists focused on MUPS-patients, the ability to reassure patients effectively is one of the most important skills to improve. In the Dutch multi-disciplinary guideline for MUPS (Trimbos-institute in co-operation with CBO, 2009) one of the applicants (Blankenstein) performed as committee-member/editor the systematic review on MUPS and reassurance.

The study question was: ‘What is the evidence for the impact of reassurance in MUPS patients on the prevention of somatoform disorders’?

Results: four relevant studies were identified. In a qualitative study of interpretation of reassurance among patients attending rheumatology clinics (Donovan & Blake, 2000) 35 patients were interviewed before and after visiting the medical specialist. Analysis of the recorded interviews showed that reassurance played an important role in the information given by the specialist. The doctors were emphasizing the early stage or the mildness of the disease and the chance on recovery. This information was not reassuring patients, but even strengthened patients' alarming cognitions about pain and invalidity. Patients who noticed that their doctor was addressing their problems felt more reassured.

The conclusion of this and other studies (Dowrick, 2004; Ring, 2004; Epstein 2007) is there are indications that doctors use often inadequate skills for reassuring, such as explanations which are not fitting the patient, medical actions and treat the symptoms of little importance.

A broad range of MeSH terms on MUPS, doctor-patient communication, normal test result, reassurance and psycho education has been performed and can be delivered on request.

TIME SCHEDULE:

Month 1-2 Preparation phase:

- research protocol, questionnaires, information materials;

- finalizing training syllabus, design, educational materials

- informational mailing to doctors

- informational conference for doctors, recruiting doctors

Month 3-20 Data collection and intervention phase:

- inclusion of doctors for intervention and control group

- inclusion of MUPS patients

- randomization of individual doctors into intervention and control condition

- pre-training group measurements by video-taping consultations and by patient questionnaires

- training intervention doctors

- post-training group measurements by video-taping consultations and by patient questionnaires

- collection of video-tapes and questionnaires

- assessment of video-taped consultations

- data-entry

- data-cleaning

Month 21-24 Analysis and report phase:

- data-analysis

- writing articles on effectiveness and economic evaluation

- final report to sponsor ZonMw

**References**

Barsky AJ, Orav EJ, Bates DW. Somatization increases medical utilization and costs independent of psychiatric and medical co morbidity. Arch Gen Psychiat 2005;62:903-10.

Cocksedge S, May C. The listening loop: a model of choice about cues within primary care consultations. Med Educ 2005;39:999-1005.

Donovan JL, Blake DR. Qualitative study of interpretation of reassurance among patients attending rheumatology clinics: ' is it a touch of arthritis, doctor?', 2000, British Medical J; 320: 541-544.

Dowrick CF, Ring A, Humphris M, Salmon P. Normalization of unexplained symptoms by general practitioners: a functional typology. British Journal of General Practice 2004;54:165-70.

Epstein RM, Hadee T, Carroll J, e.a. 'Could this be something serious?'. Reassurance, uncertainty and empathy in respons to patients' expressions of worry. Journal of General Internal Medicine 2007; 22: 1731-1739.

Howard L, Wessely S, Leese M, e.a. Are investigations anxiolytic or anxiogenic? A randomized controlled trial of neuroimaging to provide reassurance in chronic daily headache. Journal of Neurology, Neurosurgery and Psychiatry, 2005;76:1558-1564.

Maiden NL, Hurst NP, Lochhead A, Carson AJ, Sharpe M. Medically unexplained symptoms in patients referred to a specialist rheumatology service: prevalence and associations. Rheumatology (Oxford) 2003;42:108-12.

Multidisciplinaire Richtlijn Somatisch Onvoldoende verklaarde Lichamelijke Klachten en Somatoforme Stoornissen, 2009.

Trimbos-instituut en het Kwaliteitsinstituut voor de Gezondheidszorg CBO in opdracht van de Landelijke Stuurgroep Multidisciplinaire Richtlijnontwikkeling in de GGZ.

Nimnuan C, Hotopf M,Wessely S. Medically unexplained symptoms: an epidemiological study in seven specialities. J Psychosom Res 2001;51:361-7.

Peters S, Stanley I, Rose M, Salmon P. Patients with medically unexplained symptoms: Sources of patients' authority and implications for demands on medical care. Soc Sci Med 1998;46:559-65.

Petrie KJ, Muller JT, Schirmbeck F, Donkin L, Broadbent E, Ellis CJ, Gamble G, Rief W. Effect of providing information about normal test results on patients reassurance: randomized controlled trial. Brit Med J 2007;334:352.

Reid S, Wessely S, Crayford T, Hotopf M. Medically unexplained symptoms in frequent attenders of secondary health care: retrospective cohort study. Brit Med J 2001;322:767

Ring A, Dowrick C, Humphris G, et al. Do patients with unexplained physical symptoms pressurize general practitioners for somatic treatment? A qualitative study. 2004. British Medical Journal, 328: 1057.

Salmon P, Peters S, Stanley I. Patients perception of medical explanations for somatisation disorders: qualitative analysis. Brit Med J 1999;318:372-6.

Salmon P, Dowrick CF, Ring A, Humphris GM. Voiced but unheard agenda’s: qualitative analysis of the psychosocial cues that patients with unexplained symptoms present to general practitioners. British Journal of General Practice 2004;54:171-6.

Salmon P. Conflict, collusion or collaboration in consultations about medically unexplained symptoms: The need for a curriculum of medical explanation. A review. Patient Education and Counseling 2007;67:246-254.

Speckens AEM, Van Hemert AM, Spinhoven Ph, and Bolk JH. The diagnostic and prognostic significance of the Whitely Index, the Illness Attitude Scales and the Somatosensory Amplification Scale. Psychol Med 1996; 26: 1085-1090.

Stanley IM, Peters S, Salmon P. A primary care perspective on prevailing assumptions about persistent medically unexplained physical symptoms. Int J Psychiat Med 2002;32:125-40.

Terluin B. De Vierdimensionale Klachtenlijst (4DKL). Een vragenlijst voor het meten van distress, depressie, angst en somatisatie. Huisarts Wet. 1996;39:538-547.

Terluin B, van Marwijk HW, Adèr HJ, de Vet HC, Penninx BW, Hermens ML, van Boeijen CA, van Balkom AJ, van der Klink JJ,Stalman WA. The Four-Dimensional Symptom Questionnaire (4DSQ): a validation study of a multidimensional self-report questionnaire to assess distress, depression, anxiety and somatization. BMC Psychiatry. 2006;6:34.

Thomas KB. General-practice consultations - Is there any point in being positive? Brit Med J 1987;294:1200-2.

Verhaak PFM, Meijer SA, Visser AP and Wolters G. Persistent presentation of medically unexplained symptoms in general practice. Family Practice 2006; 23: 414-20.

De Waal MWM, Arnold IA, Eekhof JAH, et al. Somatoform disorders in general practice: prevalence, functional impairment, and co morbidity with anxiety and depressive disorders. British Journal of Psychiatry 2004;184:470-6.

Ware JE Jr. SF-36 health survey update. Spine. 2000;25(24):3130-9.

Zavestoski S, Brown P, McCormick S, Mayer B, D'Ottavi M, Lucove JC. Patient activism and the struggle for diagnosis: Gulf War illnesses and other medically unexplained physical symptoms in the US. Soc Sci MEd 2004;58:161-75

1. **SUMMARY OF MAJOR CHANGES TO ORIGINAL RESEARCH PROTOCOL**
2. We decided to **switch from a single center to a multi-center study** to reach the sufficient numbers of participants.
3. The **study population** contained not only medical specialists, residents and outpatients from the Erasmus MC Rotterdam. We also included doctors and patients from five other Dutch hospitals: Sint Antonius Hospital Nieuwegein; Maasstad Hospital, Rotterdam; Medical Center Haaglanden, The Hague; Albert Schweitzer Hospital, Dordrecht; Diakonessenhuis, Utrecht.
4. We started this research at 1-04-2011 and **extend the research period** with one full year till 1- 04 -2014 to reach sufficient numbers of participating doctors.
