# Supplementary material for: Training Medical Specialists to Communicate Better with Patients with Medically Unexplained Physical Symptoms (MUPS). A Randomized, Controlled Trial
Source: PLoS One. 2015 Sep 18;10(9):e0138342. doi: 10.1371/journal.pone.0138342 (PMC4575077; doi:10.1371/journal.pone.0138342)
Supplement: S3 Appendix — (DOC) [file pone.0138342.s003.doc]

##

**Code Book**

**based on the Four Habits Coding Scheme and adjusted for medical specialists in their consultations with MUPS patients**

**I. Exploration with SCEBS**

**I.1 Interested in the patient’s understanding of the problem**

**1.** The clinician makes no attempt/shows no interest in understanding the patient's perspective

Code 1 if clinician: fails to elicit the patient’s understanding of the problem; interrupts the patient when she is explaining her understanding of the problem; fails to respond to the patient's understanding of the problem; dismisses the patient’s understanding of the problem

Index:

- “So, your right hand is weak? It sounds like you had a stroke.”

Other Examples:

- P: “It’s probably my lack of sleep that is causing my toe to hurt.”

C: “There’s no reason to believe that lack of sleep can cause pain in your toe.”

- P: “I’m sure the air-conditioning caused my runny nose.”

C: “Your runny nose is likely viral. Drink a lot of juice and take some

decongestants.”

**3**. The clinician shows brief or superficial interest in exploring the patient’s understanding

of a problem

Code 3 if clinician: doesn’t directly ask, but is interested when the patient explains her understanding; asks closed-ended questions to solicit the patient’s understanding without allowing elaboration

Index:

- “So, your right hand is weak? Do you think you could’ve had a stroke?”

Other Examples:

- "Do you think the generics are not as good?”
- “Why do you think you get headaches with exercise? Could it be your sinuses?”

**5**. The clinician shows great interest in exploring the patient's understanding of a problem

Code 5 if clinician: directly asks patient what the symptoms mean to her; attempts to understand to what the patient attributes the symptoms; redirects the patient's question for the patient to answer herself

Index:

- “So, your right hand is weak? Why do you think that is?”

Other Examples:

- P: “How do you think the stroke affected my behavior?”

C: "Do *you* think the stroke affected your behavior?"

- "Why are you worried about using generics?"
- "What made you decide to stop the medication?"
- "Why are you having trouble dieting?"
- "When you say you can hear pulsating in your ears, what do you worry about?"
- "What does the numbness mean to you?"

**I.2 Shows interest in impact of symptoms on patient’s activities/behavior**

**1.** The clinician does not show interest in impact of symptoms on the patient's activities

Code 1 if clinician: does not ask the patient about impact of symptoms on activities; cuts off/discourages patient expression of activities (possibly through body language, lack of continuers); asks about activities and behavior without linking it to the symptoms; uses reflective listening, but only to limit expansion

Index:

- C: “We can increase the dose of Tylenol for the arthritis.”

P: “Doctor, I live alone and-“

C: “Like I said, the Tylenol should help.”

Other Examples:

- P: “Since I tried the Prozac I’ve been able to have more sport activities with friends.”

C: “That’s nice. Now, have you been having any problems with side effects?”

**3.** The clinician shows superficial interest in impact of symptoms on the patient's activities

Code 3 if clinician: questions the patient about impact of symptoms on personal activity with obvious lack of interest; asks the patient about activities but interrupts or signals a preference for a quick response or only briefly links to the symptoms

Index:

- C: “You live alone. Does the arthritis causes practical problems?”

P: “Yes, doctor.”

C: (interjects) “That must be difficult. I think if we increase the dose of Tylenol the pain will go away.”

**5**. The clinician shows interest in the impact of symptoms on patient's activities

Code 5 if clinician: asks the patient about impact of symptoms on behavior, work, daily activities; recalls past activities while inquiring about current issues; uses open-ended questions to inquire about impact of symptoms on patient’s activities; actively/with interest listens to the patient.

Index:

- C: “You live alone. Do you have any difficulty during the day because of your arthritis?”

P: “Yes, doctor. It’s hard for me to cook because I can’t open jars and it’s hard to take the plates down from the pantry.”

C:”That must be frustrating. Especially because I know you love to cook dinners for your family. I suggest you see a occupational therapist

**I.3 Shows interest in impact of symptoms on patient’s social environment**

**1.** The clinician does not show interest in the impact of symptoms on patient's social environment

Code 1 if clinician: does not ask the patient about impact of symptoms on patient’s social environment; cuts off/discourages patient expression of issues concerning the social environment (possibly through body language, lack of continuers); uses reflective listening, but only to limit expansion

Index:

- C: “We can increase the dose of Tylenol for the arthritis.”

P: “Doctor, I live alone and-“

C: “Like I said, the Tylenol should help.”

Other Examples:

- P: “Since I tried the Prozac I’ve been able to have closer friendships.”

C: “That’s nice. Now, have you been having any problems with side effects?”

**3.** The clinician shows superficial interest in impact of symptoms on the patient's social environment

Code 3 if clinician: questions the patient about impact of symptoms in patient’s social environment with obvious lack of interest; asks the patient about social environment issues but interrupts or signals a preference for a quick response

Index:

- C: “You live alone. Does your manager know of your arthritis?”

P: “No, doctor.”

C: (interjects) “I’m surprised! I think if we increase the dose of Tylenol the pain will go away.”

**5**. The clinician shows interest in impact of symptoms on the patient's social environment

Code 5 if clinician: asks the patient about influence of symptoms on family, colleagues, relationships; recalls past social environment issues while inquiring about current issues; uses open-ended questions to inquire about social environment; actively/with interest listens to the patient talk about social environment

Index:

- C: “You live alone. Are you able to speak about the arthritis with your colleagues?”
- P: No doctor, I have the feeling I am already bothering my company with my sick-leaf. I don’t want to give more troubles.
- C: That must be difficult. Perhaps you should discuss this with the occupational health service?

C: “Is there anyone in your family who lives nearby and could help?”

**I.4 Encourages expression of emotions related to the symptoms**

**1.** Clinician clearly discourages, shows no interest in or cuts off the patient’s expression of

emotion related to symptoms of patient

Code 1 if clinician: signals verbally or nonverbally that it is not okay to express emotions

Index:

- P: “It really scares me that my blood pressure is so high.”

C: “There’s nothing to be scared of. We should just increase the dosage.”

Other Examples:

- “I recommend surgery. There’s no reason at all to be scared or worried.”
- “I thought we talked about your worries last time.”
- “Worrying will only make things worse.”

**3.** Clinician shows little interest or encouragement for the patient's expression of emotion

Code 3 if clinician: is not fully attentive when the patient is expressing emotion (i.e., reading chart, writing, checking pager); prompts the patient to express her emotions, but with obvious lack of interest (i.e., yes/no questions, unemotional tone of voice)

Index:

- P: “It really scares me that my blood pressure is so high.”

C: “Why?” (writing in chart)

Other Examples:

- “Are you worried about the operation?”
- “You would feel better if you had some help at home, right?”

**5**. Clinician encourages the patient to express emotion related to the symptoms of patient and/or is openly receptive to patient's expression of emotion

Code5 if clinician: signals verbally or nonverbally that it is okay to express feelings related to the symptoms; poses f. e. open-ended questions addressing emotional issues; attentively listens to the patient’s expression of emotion (i.e., open body language and continuers); appears to show empathy for the patient’s feelings

Index:

- P: “It really scares me that my blood pressure is so high.”

C: “It can be a frightening thing. What scares you about it?”

P: “My father had high blood pressure all his life and eventually his heart stopped.”

Other Examples:

- "Does the dizziness ever frighten you?"
- "So when the headaches come back, how does that make you feel?”

**I.5 Explores physical symptoms**

1. Clinician doesn’t explore the physical symptoms properly and doesn’t perform physical examinations at all.

Code 1 if clinician: explores some but not all of the physical symptoms and performs no other examinations then watching from behind its desks and summarizing the data from the electronic patient files/referral letter.

Index

* C:”I see that you already suffer 2 years from lower back pain and that all the research has been done. I see no need in doing more medical investigations”.

* C:”In my opinion your tiredness is nothing else then the result of overweight and therefore I see no reason to do additional explorations or tests myself.

3. Clinician explores somatic symptoms without proper physical examinations.

Code 3 if clinician: explores some but not all of the physical symptoms by asking when they occur, where, for how long, with what kind of pain etc and performs limited examination (blood pressure, weight, tongue).

Index

- C:”Can you tell me exactly where the pain is located”?
- C:”We don’t have time for all your concerns therefore I suggest to start with the pain in your left leg”.
- C:”I can see that you lost weight. Do you know how much your weight is at the moment”?

5 Clinician explores somatic symptoms seriously and also performs physically examination.

Code 5 if clinician: explores the physical symptoms properly by asking which symptoms occur, when, where, how long, with what kind of pain etc and performs physical examinations. If the symptoms are too many for the limited time of the consultation the doctor mentions that explicitly and makes a proposal how to deal with that in time.

Index

- C:”Tell me more about your stomach pain”.
- C:”Can you describe to me what kind of pain it is you feel when your belly hurts”?
- C:”I know like to examine your stomach myself, so if you want to take place in the examination room…”.

**I. 6 Acknowledges the reality of patient’s symptoms**

**1**. Clinician makes no attempt to respond to/validate the reality of patient’s symptoms

Code 1 if clinician: belittles or challenges the patient's reality of symptoms is judgmental; ignores patient's reality of symptoms, denies the existence of certain symptoms

Index:

- P: “Even just thinking about the possibility that it’s cancer scares me.”

C: “There’s nothing to be scared of. We’ll schedule the test for Monday.”

Other Examples:

- P: I have also very painfull legs at night time and I’m not able to carry my shopping bags anymore.

C: We concentrate on your headache right now.

- P: “Will that test hurt at all?”

C: “Just bring this prescription to the lab and you’re all set.”

**3.** Clinician may briefly acknowledge the patients' reality of symptoms, but fails to offer

acceptance/validation

Code 3 if clinician: responds to the patient’s reality of symptoms in a way that stifles further expression

Index:

- P: “Even just thinking about the possibility that it’s cancer scares me.”

C: “I know you are scared, but I am sure that things will work out.”

Other Examples:

- "Yup, it'll drive you crazy."
- "I know that your symptoms can be very bothersome, but you have to try to carry on regardless."

**5.** Clinician makes comments clearly indicating acceptance/validation of patient's reality of symptoms.

Code 5 if clinician: acknowledges the appropriateness of the patient’s reality of symptoms; identifies with patient's situation

Index:

- P: “Even just thinking about the possibility that it’s cancer scares me.”

C: “Of course. Anybody would feel scared at the thought of cancer. It’s a frightening thing.”

Other Examples:

- "I know facing the pains in your shoulder and knees isn’t easy, but you have to give yourself credit for losing weight."
- P: “I mean, they’ve done all kinds of tests & can’t find anything wrong. Maybe I’m crazy.”

C: “It’s easy to doubt yourself when no one can find proof that you’re in pain, but if you feel it I’m sure it’s real.”

**II Informing MUPS patients about findings and explaining perpetuating factors**

**II.7 Summarizes information according all SCEBS items using patient's perspective**

1. Code 1 if clinician doesn’t mention only the somatic aspect of the complaints of the SCEBS-items from the exploration
2. Code 2 if clinician mentions 2 of the SCEBS items
3. Code 3 if clinician mentions 3 of the SCEBS-items
4. Code 4 if the clinician mentions 4 of the SCEBS-items
5. Code 5 if clinician mentions all 5 SCEBS-items of the patient’s symptoms.

**II.8 Frames information in positive language**

**1.** Clinician frames diagnostic and relevant information in **negative terms**

Code 1 if clinician: only tells the outcome ‘that there has been no results’, ‘there’s nothing to worry about’, we don’t have to do this or that.

Index:

- P: “I’m concerned about my blood pressure being so high. It makes me tired all the time and I can’t do all the things I used to be able to.”

C: “I’ve examined you and we don’t have to control your bloodpressure. There is nothing I could find to worry about.

Other Examples:

- P: “Will I gain weight if I stop smoking?”

C: “When you stop smoking, other problems can occur. We’ll monitor your health as much as we can to see that no serious problems occur.”

**3.** Clinician makes cursory attempts to frame diagnostic and relevant information in **neutral terms and addressing some of the patients concerns**

Code 3 if clinician: addresses the patient's concern(s)(possibly addressing the patient’s concerns at the end of the visit while standing to exit the room); emphasizes an issue outside of patient's range of concern

Index:

C: “there’s no doubt that your family history is relevant, your tiredness might have to do with some of your problems; the blood results were as I had expected. I think you should focus more on….”

Other Examples:

- “Ok, we went over the prescriptions... oh, you were concerned about gaining weight. Try snacking on rice cakes. They’re low calorie. Good luck.”

**5.** Clinician summarizes diagnostic and relevant information in ways that reflect the patient's initial presentation of concerns in **positive language.**

Code 5 if clinician: uses the patient's frame of reference and/or actual words when presenting information

Index:

- P: “I know you were concerned about your heart. I just examined you and I felt a regular heartbeat and your blood pressure is all right.

Other Examples:

- “It’s clear that the most important concern you have about your knee is how soon you can be playing soccer again; in the examination I felt a proper muscle tone and therefore I can reassure you that your knee is able to recover within a few weeks.

**II.9 Explains symptoms are not caused by disease**

1. Clinician tells that the symptoms are not a disease in his specialty without explaining why, or doesn’t mention that symptoms are not a disease, or tries to explain symptoms by minor physical abnormalities

Code 1 if clinician: just tells that the symptoms are nothing to worry about

Index:

C: Your shoulder is not broken, there is no need for worriness.

C: The headache is not caused by a brain tumor and that’s what you were thinking.

3 Clinician tells symptoms are no disease within his specialty and explains to the patient why without making connection to the patient’s perspective

Code 3 if clinician: explains the symptoms are no disease without using patients perspective

Index:

P: I’m afraid my skin problems are shingles.

C: Your symptoms luckily don’t fit that diagnosis nor that of cancer. The lab results would show a different figure, higher liver scores too.

C: Your lung capacity got better since the last visit. That’s good news and it means your difficulties in walking up the stairs are not caused by lung disease..

**5.**clinician tells clearly symptoms are not a disease within his specialty and explains clearly why

Code 5 if clinician: explains the nature of symptoms are not a disease and is able to explain clearly to the patient why**.**

Index:

C: Your lungs are healthy for their age and there is absolutely no asthma. I will explain you why. Look here is a picture of the lungs, do you see how ……….

**II.10 Explains perpetuating factors**

1. Clinician tells that there is no medical explanation for the symptoms and that the patient has to live with them, or talks like there is a medical explanation, or says a medical explanation is still to be found (without mentioning MUPS).

Code 1 if clinician: is not able to explore the nature of the MUPS, says it is not to be explained.

Index:

P:”So you didn’t find anything?”

C:”That’s right, I can’t find a reason for your stomach pain. You are having these symptoms for a several years now and I think you have to live with them. I can give you some painkillers for when the pain gets very heavy at night. You can take two of them.

3. Clinician explains the nature of MUPS and tells that a lot of people have unexplained symptoms and that it is a very normal situation.

Code 3 if clinician: convinces the patient that there is nothing to worry about and that there are a lot of symptoms without medical explanation.

Index:

C:”Based on these test results I can assure you that there is no brain tumor. We do not know why your headaches are so frequently occurring. Actually, I can tell you that I see a lot of women here who have similar symptoms like you and most of the time the symptoms disappear after a while. It surely is important to watch your daily patterns and therefore I think you can benefit from a consultation at our psychologist.

5. Clinician tells that a lot of people have symptoms without having a disease and that they are able to influence the symptoms and gain more quality lifetime.

Code 5 if clinician: explains that we often do not know the medical reason for symptoms but we do know there are factors that influence the perpetuating and worsening of symptoms.

Index:

C: “Your ears look normal and healthy. There is no medical explanation for your symptoms. Your pain is real, but as in many other cases, we don’t know what causes the pain. What we do know is that your symptoms have impact on your life and that it

is helpful to discover which factors make your symptoms worse and how you can influence them so that you can have a better life”

C:”I am sure you don’t have a disease and therefore I would like to explore with you what influences your feeling of tiredness. You told me that you have good days and bad days. Most people in my practice discover that they can deal with their symptoms in an better way if they know what makes their days worse or happy. For example playing with the children before bed-time makes the pain less present.

# II.11 Uses drawings in the explanation of MUPS

**1.** Information is stated in ways that are technical or above patient's head and /or no use of drawings is made.

Code 1 if clinician: consistently uses unnecessary technical words without clarification

Index:

- “The artery is not occluded. That is when plaques have built up its hard for the blood supply to meet the demands of the heart when you exercise. That’s why you get angina. In your case your heart condition is all right and you don’t have to worry at all.”

Other Examples:

- “This test will indicate whether or not your megaloblastic anemia is due to folate deficiency.”

**3.** Information contains some jargon and is somewhat difficult to understand; the drawings were too complicated

Code 3 if clinician: consistently uses unnecessary technical words that may or may not be explained and drawing doesn’t support the explanation (makes it even more complicated)

Index:

- C:”As I see it your symptoms get more and more and also worse. Just like a vicious circle, look here it goes like this. You are afraid of back pain and therefore you don’t work in the garden anymore, which you loved to do. That makes you unhappy and you feel depressed and because of that you collapse and are very much focused on what hurts. You know what I am telling, don’t you?”

**5.** Information is stated clearly, with little or no use of jargon and with use of drawings

Code 5 if clinician: uses language appropriate to the patient’s level of understanding; explains the meaning of technical words; or uses technical words without explanation, but the patient is obviously familiar with these words. Draws a vicious circle, star, spiral or a fish-bone to illustrate the explanation of MUPS.

Index:

- C:“You told me that you have already for some years lower back pain. You also said that in the last year you lost your job and that the pain is getting worse and that you are not able to do the household anymore. So over time there are a lot of things which as a result of the back pain get worse. You are going downhill. .
- P:”That is right, can you explain more about it..
- C: It’s just like a downward spiral, I will draw one for you….”

**II. 12 Acknowledges perspectives of patient concerning symptoms and treatment options**

**1.** Doctor doesn’t mention information from patient’s and patients expectations perspective (items 1 to 6 from section I).

Code 1 if clinician: just explains his own theory without mentioning the perspectives of the patient.

Index:

* P : “I think my kidneys are damaged because of the work I did in the chemical factory and I am afraid that I will soon die, just as my best friend, who had exactly the same symptoms as I do. That’s why my back is sore and I am not able to work and I really want that MRI .

* C:”What we see is your kidneys work fine.I suggest you take more rest in the afternoon and drink less alcohol. I like to see you in two months and guess that your levels will be much better by then.

3. Doctor partly mentions information from the patients perspective

Code 3 if clinician: informs patient without explicitly mentioning that it might be worrying to hear that there is no medical explanation for symptoms.

Index:

P: “I think my kidneys are damaged because of the work I did in the chemical factory and I am afraid that I will soon die, just as my best friend, who had exactly the same symptoms as I do. That’s why my back is too painful to work. I want you to check me with a MRI.

C: “Your back pain has nothing to do with your kidneys. I can assure you based on the lab results that you don’t need to worry. Your kidneys are just working fine.

P: “That doesn’t convince me, why can I have such great pain in my back? I saw it with my own eyes how my friend died. I want a MRI done.

C: “You are upset by your friends death, but I don’t see a medical reason to schedule you for a MRI.

5. Doctor mentions information from the patients perspective effectively

Code 5 if clinician: reflects on perspectives for patient effectively

Index:

P: “I think my kidneys are damaged because of the work I did in the chemical factory and I am afraid that I will die soon, just as my best friend, who had exactly the same symptoms as I do. My homoeopathist said it’s definitely something in my kidneys and that you must run some serious test.

C: “I’ve heard you are concerned that your kidneys are damaged due to chemicals in your work as was the case with your friend and that you and your homoeopathist think that’s the cause of your back pain. That’s clear to me and why I’ve done these examinations.

**II. 13 Explains perspectives of doctor concerning symptoms and treatment options**

**1.** Doctor doesn’t mention his own perspectives and ideas

Code 1 if clinician: doesn’t explain his own theory about symptoms and treatment

Index:

* P : “I think my kidneys are damaged because of the work I did in the chemical factory and I am afraid that I will soon die, just as my best friend, who had exactly the same symptoms as I do. That’s why my back is sore and I am not able to work and I really want that MRI .

* C. With less salt in your diet you should feel better soon.

3. Doctor mentions his perspective on patient’s symptoms and treatment partly

Code 3 if clinician: explains his ideas about symptoms and treatment in a way that is too medical or shortly to grasp it for the patient

Index:

C: “I have examined your kidneys, liver and blood. Negative results on all the tests we did! ; lowering the medication is the best treatment now.

5. Doctor mentions his perspective on the patient’s symptoms and treatment very clearly to the patient

Code 5 if clinician: explains his perspective in a very understandable way

Index:

C: Your symptoms are not caused by kidney failure. What we often see is that in your age kidneys work slower. That is no problem. I will explain it in this way (shows pictures of kidneys…). We can support the functioning of the kidneys with medication and a diet. My experience is that most patients feel much better after a month. A MRI would give me no other information.

**II. 14 Connect perspectives of doctor AND patient**

**1.** Doctor doesn’t connect information from patient’s perspective and doctors ideas about symptoms and treatment

Code 1 if clinician: just repeats his theory or patients theory (ships passing in the night f.e.) .

Index:

* P : “I think my kidneys are damaged because of the work I did in the chemical factory and I am afraid that I will soon die, just as my best friend, who had exactly the same symptoms as I do. That’s why my back is sore and I am not able to work and I really want that MRI .

* C:”What we often see is that in your age the kidneys are working slower. That is no problem. That can be genetic, but we don’t really know. I suggest you take more rest in the afternoon.

3. Doctor partly connects his perspective with that of the patient

Code 3 if clinician: connects partly his ideas with the patient’s ideas about symptoms and treatments

Index:

P: “I think my kidneys are damaged because of the work I did in the chemical factory and I am afraid that I will soon die, just as my best friend, who had exactly the same symptoms as I do. That’s why my back is too painful to work. I want you to check me with a MRI.

C: “I hear you are afraid of serious kidney diseases but I can assure you based on the lab results that you don’t need to worry. Your kidneys are just working fine. You are shocked by your friends death, but I don’t see a medical reason to schedule you for a MRI.

5. Doctor connects his perspective with the patient

Code 5 if clinician: reflects on differences and agreements between theories of patient and doctor concerning symptoms and treatment

Index:

P: “I think my kidneys are damaged because of the work I did in the chemical factory and I am afraid that I will die soon, just as my best friend, who had exactly the same symptoms as I do. My homoeopathist said it’s definitely something in my kidneys and that you must run some serious test.

C: “I’ve heard you are concerned that your kidneys are damaged due to chemicals in your work and that you and your homoeopathist thinks that’s the cause of your back pain. That’s clear to me and that’s why I’ve done these examinations. Unlike you I don’t think your back pain has to do with your kidneys. They function properly. It’s my experience that more people suffer from back pain without having any disease like kidney failure. Is that worrying you, to have pain without a disease? I guess it is something you didn’t expect at all. Is it?

**II.15 Allows time for information to be absorbed**

**1.** Clinician gives information and continues on quickly without allowing the patient an

opportunity to absorb

Code 1 if clinician: offers a quick "ok" without a pause after information is given; quickly moves from topic to topic without pausing in between; ignores the patient’s attempt to absorb the information by asking a question; speaks very quickly

Index:

- C: “If you take this once at night you won’t have pain when you urinate. Ok? Now about your blood pressure…”

Other Examples:

- “For the diabetes here’s a prescription for glyburide. The cough could be pneumonia so here’s a prescription for antibiotics, and try to get the blood work done before I see you next week. All right, take care.”

Upon a patient's interjection, "We can talk about this more later.”

**3.** Clinician briefly allows for the patient to react, but then quickly moves on

Code 3 if clinician: offers an “ok?” after most information is given, but doesn’t pause long enough to allow patient absorption; minimally responds to the patient’s attempt to absorb the information by asking a question

Index:

- C: “If you take this once at night you won’t have pain when you urinate. Ok?” (pause)

P: “(thinking) “I only take this once and then I can pee without a problem, right?”

C: “Right. Now, about your blood pressure…”

Other Examples:

- P: “So it was my hip and not my knee causing the pain?”

C: “Yup. Now here’s the prescription for ibuprofen.”

**5.** Clinician gives the patient time and opportunity to react to and absorb information

Code 5 if clinician: pauses prior to shifting topics; deliberately offers an "ok?" with a pause after presenting information; speaks at a pace which allows the patient to grasp the information and to interject questions

Index:

- C: “If you take this once at night you won’t have pain when you urinate. Ok?” (pause)

P: (thinking) “I only take this once and then I can pee with no problem, right?”

C: “That is absolutely right. There should be no pain & no hesitancy.” (pause)

Other Examples:

- C: “The pain and swelling in your foot is probably an infection due to diabetes. We’ll try some antibiotics while we wait for the lab tests.” (pause)

P: “MmHmm. I think I understand. In case it’s an infection I’ll take antibiotics now.”

- C: “That’s right.” (pause)

#

##### III Follow up and making appointments

# III.16 Explains rationale and possible outcomes of test results prior to testing

**1**. Clinician offers/orders tests, giving little or no rationale for these nor discusses prior to testing the meaning of possible outcomes

Code 1 if clinician: simply states that a test/procedure should be done without explanation; fails to warn the patient about side-effects

Index:

- “You should have a CT scan done.”

Other Examples:

- “Come over to the table and I’ll do a neurological exam.” (proceeds without further explanation)

**3.** Clinician only briefly explains the rationale for tests and mentions possible outcomes shortly.

Code 3 if clinician: minimally explains the reason for a test/procedure and eventually mentions possible outcomes

Index:

- "A CT scan would be better than an MRI to look at your jawbone. When the CT shows normal results then it means I fully can reassure you there is no disease.”
- “We are going to have your electrolytes tested because of what the EKG showed.”

**5.** Clinician fully/clearly explains the rationale/results of future test and discusses the possible outcomes before testing

Code 5 if clinician: thoroughly explains the reasons why certain tests are necessary and how a positive of negative outcome will look like and what that means for follow-up

Index:

- "In your case a CT scan would actually be better than an MRI and it has nothing to do with money or cost. An MRI would miss what we suspect may be most important, to take a look at your jawbone. An MRI does not take good pictures of bone, but this is exactly what a CT scan does best. When the CT shows something extraordinary I will make a punction and do further research. If the CT shows normal results then it means I fully can reassure you there is no disease.”

Other Examples:

- “If the punction shows X (doctor illustrates with an example or drawing) then it means your symptoms are not caused by a disease. Do you understand that? Do you have any further questions before we start the punction?”
- “In order to find out what is causing your tiredness we should get two tests done. The first one will show whether you have enough vitamin B_12_, which can cause anemia, and the other will show whether you have enough folate, another vitamin, which can cause this type of anemia too. Then we can make sure we’re treating you properly.”

**III.17 Effectively tests for comprehension**

**1.** Clinician makes no effort to determine whether the patient has understood; doesn’t ask the patient whether he/she understands; seems unconcerned if patient gives indication of not having understood

Code 1 if clinician: provides information without attempting to determine whether the patient has understood

Index:

- C: “So the best thing to do would be to increase you Synthroid dosage. Now, about your cholesterol…”
- C: :”Is that clear?”

P: “Uh, not really.

- C: “Well, the important thing is that you just keep taking your medications as prescribed.”

**3.** Clinician briefly or ineffectively tests for the patient's comprehension

Code 3 if clinician: briefly responds to the patient’s attempts at clarification; moves on before the patient has clearly understood

Index:

- C: “Do you understand why we’re increasing the Synthroid dosage?”

P: “Not exactly. Is it because I’m gaining weight?”

C: “No. Your thyroid hormone levels are too low. And now about your cholesterol…”

Other Examples:

- C: “Is that clear?”

P: “Not really.”

C: “Well, then let me repeat that.” (Restates with little change or elaboration)

**5.** Clinician effectively tests for the patient's comprehension of information presented

Code 5 if clinician: deliberately seeks to find out if the patient understands the information presented; encourages the patient to ask for clarification of information

Index:

- C: “Do you understand why we’re increasing the Synthroid dosage?”

P: “Not exactly. Is it because I’m gaining weight?”

C: “You’re gaining weight because you don’t have enough thyroid hormone in your body. We’re increasing the dosage because we need to bring the level of thyroid hormone up just a little. Does that make more sense?”

P: “Oh, I see.”

Other Examples:

- “Do you have any questions about what I’ve just said?”
- “What would you say at home, when they ask what did the doctor tell you”?
- “Can you repeat that back to me to see if I’ve made things clear.”

“I hope this is making sense.” (pause for patient response)

**III.18 Encourages involvement in decision-making**

**1.** Clinician discourages/ignores the patient's efforts to be part of decision-making process; doesn’t encourage the patient to make the decision about the next step together

Code 1 if clinician: fails to acknowledge the patient’s stated preference; quickly proceeds through the decision-making process without considering/asking the patient’s opinion; openly discourages the patient’s input

Index:

- C: “We’ll start you on Prozac to help with the depression.”

P: “Ya know, I tried the Prozac before and I had problems…uh… getting an erection. Can I try something else?”

C: “Prozac is the best for you.”

Other Examples: “You shouldn’t believe everything you see on TV and in the papers. You can trust my judgment about what works best.”

**3**. Clinician shows little interest in encouraging the patient's input into the decision-making

process, or responds to the patient's attempts to be involved with relatively little enthusiasm

Code 3 if clinician: inconsistently attempts to involve the patient in the decision-making process; acknowledges the patient’s input but does not negotiate

Index:

- C: “We can start you on Prozac to help with the depression.”

P: “Ya know, I tried the Prozac before and I had problems…uh…getting an erection. Can I try something else?”

C: “A lot of people have that problem with Prozac. I think you should give it another try, and if that doesn’t work we’ll try bupropion.”

Other Examples:

- “I know you don’t want to have the test done before your vacation, but you should.”
- “There are various ways to treat this that we can try, but my preference is…”

**5.** Clinician clearly encourages the patient's input into the decision making process

Code 5 if clinician: makes decisions a joint effort that includes patient input; acknowledges and supports (if appropriate) patient’s decisions about treatment

Index:

- C: “We can start you on Prozac to help with the depression.”

P: “Ya know, I tried Prozac before and I had problems…uh…getting an erection. Can I try something else?”

C: “Ok. A lot of people have that problem with Prozac. Let’s consider some other options that made not be as troubling for you.”

Other Examples:

- “Do you think that the Verapamil, once a day, would be easier for you to take?”
- “We can do that now or later, whichever is better for you.”
- “What do you prefer: that I give you advice about the preferred treatment or that I give you the various options so that you can choose
- P: “I know I’m being a pain asking so many questions.”

C: “No, I’m quite pleased to have you informed so we can make good decisions for you.”

**III.19 Explores acceptability of treatment and/or follow-up plan**

**1.** Clinician offers recommendations for treatment with little or no attempt to elicit the patient's

acceptance of (or likelihood of following) the plan; disregards or dismisses patient’s concerns for compliance.

Code 1 if clinician: quickly offers recommendations without attempting to determine the patient’s potential for compliance

Index:

- C: “I think we’ve covered everything. So, I think this is what we came up with. You’ll contact the physical therapist tomorrow, start taking the anti-inflammatory and stay off the soccer field until the physical therapy is done. I’ll see you next time.”

Other Examples:

- “Ok, so that’s what you should do. Good luck with everything.”
- “Don’t be so negative. It’s not as hard as it sounds.”

**3.** Clinician makes a brief attempt to determine acceptability of the treatment plan, and moves

on quickly

Code 3 if clinician: briefly attempts to determine acceptability of recommendations; interrupts the patient’s expressed concerns about the treatment plan and/or offers hollow reassurance

Index:

- P: “So you’ll have to start checking your blood for sugar, to exercise more, and to watch your diet. Do you, think you can manage that?”

C: “I’m not so sure…”

P: ”I know it sounds tough, but I have lots of confidence in you.”

Other Examples:

- “That sounds good to you, right?”
- “So now, you’ll try to do all that before we meet next week, okay?”

**5.** Clinician explores acceptability of treatment plan, expressing willingness to negotiate

Code 5 if clinician: clearly asks the patient if they plan to follow through with recommendations; deliberately questions the patient to determine the acceptability of recommendations

Index:

- “I think this will work, but if it doesn’t sound good to you we probably ought to modify it or figure out another plan.”

Other Examples:

- “Are you willing to give it a try?”
- “You know that this could cause drowsiness, How much of a problem would that be for you?”
- “Do you think that you can do that?”

**III.20 Explores barriers to implementation of treatment and/or follow-up plan**

**1.** Clinician does not address whether barriers exist for implementation of the follow-up

Code 1 if clinician: fails to address hurdles to patient compliance to the follow-up plan

Index:

- C: “It would be best if you could try to stay on a low-fat diet. That will keep you cholesterol down.”

Other Examples:

- “I’ll switch you to the new acid-blocker and that should take care of the problem.”

**3.** Clinician briefly explores barriers to implementation of the follow-up plan

Code 2 if clinician: briefly attempts to address problems that may limit compliance

Index:

- C: “It would be best if you could try to stay on a low-fat diet. Do you think you could do this?”

P: “Well, it’s hard because when I work, I eat in the cafeteria and the foot is pretty greasy. There are no other places nearby to eat.”

C: “You really should find another place to eat lunch.”

Other Examples:

- “So we’ll switch you to the new acid-blocker. It’s supposed to be really good. Will it be a problem to switch?”

P: “My insurance only covers certain medications. I don’t know if this one is covered.”

C: “I’m sure it will be fine.”

**5.** Clinician fully explores barriers to implementation of the follow-up plan

Code 3 if clinician: thoroughly addresses a specific or unique problem that could prevent the patient from following through with recommendations; initiates discussion of barriers to patient compliance

Index:

- C: “It would be best if you could try to stay on a low-fat diet. Do you think you could do this?”

P: “Well, it’s hard because when I work, I eat in the cafeteria and the foot is pretty greasy. There are no other places nearby to eat.”

C: “Could you maybe make your lunch at home? It would take some extra time, but it would be worth it for your health.”

P: “I could try that. It might be hard each night to find time though. I’ll try.”

Other Examples:

- “Will you be comfortable wearing the brace all day?”
- “How are you planning to keep track of which pills are which?”
- “Is the co-pay for that medication ok for you?”
- “Is the aspirin upsetting your stomach too much?
- “How is your house laid out? Can you get around with a walker?”
- “Is there anyone who can help you get to the orthopedic appointment during the week?”

**III.21 Summarizes plans for follow-up**

**1.** Clinician makes no reference to follow-up plans

Code 1 if clinician: does not mention follow-up plans or the next appointment.

Index:

- “So you’re all set. Take care.”

Other Examples:

- “It was nice to meet you. Good luck.”

**3.** Clinician makes references to follow-up, but does not make specific plans

Code 3 if clinician: makes vague references to follow-up; gives the patient conflicting plans for follow-up without clarifying

Index:

- "Give me a call if you have other questions."
- “I’ll call Dr. Hopkins.”
- You came here for your back pain and from the test it’s clear that your kidneys aren’t damaged so that’s what I’ll write to your GP

**5.** Clinician makes clear and specific plans for follow-up and summarizes and makes them specified, measurable, acceptable and reasonable.

Code 5 if clinician: clearly indicates what the patient can expect after this visit; attempts to set a definite date for the next visit and/or makes a clear summary of questions, findings and answers.

Index:

- "So I’ll see you again in six weeks. That should give the anti-depressant enough time to start working and we can see how you’re doing then. You can make an appointment with the nurse before you leave the ward."
- "Keep on with the current medications and I will see you in another year for your annual, unless anything else comes up; I will also inform your GP that your lungs didn’t get worse as you and your GP were afraid of and that I did a lung function test and chose for adjustment in the medication to support your breath at nighttime more sufficiently; I also recommend you should do some relaxation therapy like yoga so you can improve your breathing."
- "Let me talk to Dr. Hopkins first and then I'll give you a call next Friday morning to schedule our next appointment."

**III.22 Displays effective nonverbal empathy in the whole consultation**

**1.** Clinician's nonverbal behavior displays lack of interest and/or concern and/or connection

Code 1 if clinician: uses little or no eye contact, bored tone of voice and/or non-attentive body language

Index:

Other examples:

- Clinician rarely makes eye contact; spends most of time with head in the patient record
- Clinician faces away from patient leaning over desk
- Clinician rests his head on his hand
- Clinician ignores the tears of a patient by concentrating on the computer
- Clinician sits upright in a removed, judgmental posture (possibly with arms folded or shoulders raised)
- Clinician answers several phone calls/

**3**. Clinician's nonverbal behavior displays neither great interest nor disinterest

Code 3 if clinician: displays inconsistent body language and/or attentiveness; seems interested in the patient but is tired or preoccupied

Examples:

- Clinician begins the interview with real interest which fades as the interview goes on
- Clinician answers a phone call/page while the patient is expanding, but otherwise shows interest in the patient
- Clinician appears very interested at some points and disinterested at other points (looking away, writing, reading)

**5.** Clinician displays nonverbal behavior that expresses great interest, concern and

connection throughout the visit

Code 5 if clinician: focuses on the patient using good eye contact; responds to patient with attentive body posture; uses receptive body posture

Examples:

- Clinician leans forward when patient speaks
- Clinician squarely faces the patient
- Clinician maintains eye contact when patient is talking, especially when discussing emotional issues
- Clinician does not cross arms or appear distracted by the chart, notes or pages
- Light touch indicating compassion and support
- Clinician’s body language/facial expression changes in response to the patient
