# Supplementary material for: Training Medical Specialists to Communicate Better with Patients with Medically Unexplained Physical Symptoms (MUPS). A Randomized, Controlled Trial
Source: PLoS One. 2015 Sep 18;10(9):e0138342. doi: 10.1371/journal.pone.0138342 (PMC4575077; doi:10.1371/journal.pone.0138342)
Supplement: S4 Appendix — (PDF) [file pone.0138342.s004.pdf]

Algemene gegevens / General Information

Programma / Programme	:	
Subsidieronde / Subsidy round	:	**Ronde 11 - Grant application VEMI**
Projecttitel / Project title	:	**Effectiveness and efficiency of a consultation skills training programme for medical specialists focused on patients with medically unexplained physical symptoms (MUPS).**
Projecttaal / Project language	:	**Nederlands / Dutch**
Geplande startdatum / Planned start date	:	**01-11-2010**
Geplande duur / Planned duration	:	**24 maanden / months**
Datum indienen / Date of application	:	**02-02-2010**
Projecttype / Project type	:	**Strategisch onderzoek / Strategic research**
Vervolg eerder ZonMw-project / Continuation previously funded project ZonMw	:	**Nee / No**

Aanvrager / Applicant

Drs. A Weiland
T: 0107034562 | *F:* | *E:* a.weiland@erasmusmc.nl

Erasmus MC
Interne Geneeskunde
Postbus 2040
3000 CA ROTTERDAM

Projectleden / Project members

Dr. L.R. Arends (Begeleider)
Functie / Position: Biostatistician | *Opleiding / Education:* WO
Studierichting / Subject: Econometry and Psychology
T: 010-4088667 | *F:* | *E:* arends@fsw.eur.nl

Erasmus Universiteit Rotterdam
Faculteit der Sociale Wetenschappen
Instituut voor Psychologie
Postbus 1738
3000 DR ROTTERDAM

Dr. A.H. Blankenstein (Begeleider)
Functie / Position: Senior-researcher and GP | *Opleiding / Education:* WO
Studierichting / Subject: Medicine
T: 020-4448198 | *F:* | *E:* ah.blankenstein@vumc.nl

VU Medisch Centrum
EMGO Institute for Health and Care Research
Huisartsgeneeskunde
Van der Boechorststraat 7
1081 BT AMSTERDAM

Dr. A.M. van Dulmen (Projectadviseur)
Functie / Position: Senior-researcher | *Opleiding / Education:* WO
Studierichting / Subject: Clinical Psychology
T: 030 - 2729703 | *F:* 030 - 2729729 | *E:* s.vandulmen@nivel.nl

Nederlands Instituut voor onderzoek van de gezondheidszorg (NIVEL)
Postbus 1568
3500BN UTRECHT

Prof. dr. E.J. Kuipers (Bestuurlijk verantwoordelijke)
Functie / Position: Prof. and Head of department Internal Medicine | *Opleiding / Education:* WO
Studierichting / Subject: Medicine
T: 010 - 7034681 | *F:* 010 - 7034682 | *E:* e.j.kuipers@erasmusmc.nl

Erasmus MC
Interne Geneeskunde
Postbus 2040
3000 CA ROTTERDAM

Prof. dr. H.T. van der Molen (Mede aanvrager)
Functie / Position: Dean Faculty Social Sciences | *Opleiding / Education:* WO
Studierichting / Subject: Clinical Psychology
T: 010 4082065 | *F:* | *E:* vandermolen@fsw.eur.nl

Erasmus Universiteit Rotterdam
Faculteit der Sociale Wetenschappen
Instituut voor Psychologie
Postbus 1738
3000 DR ROTTERDAM

Prof. dr. J.L.C.M. van Saase (Projectleider en penvoerder)
Functie / Position: Professor Internal Medicine | *Opleiding / Education:* WO
Studierichting / Subject: Medicine
T: 010 - 7034382 | *F:* | *E:* j.vansaase@erasmusmc.nl

Erasmus MC
Interne Geneeskunde
Postbus 2040
3000 CA ROTTERDAM

Drs. A. Weiland (Uitvoerder)
Functie / Position: Researcher | *Opleiding / Education:* WO
Studierichting / Subject: Adult education
T: 010 - 7034562 | *F:* | *E:* a.weiland@erasmusmc.nl

Erasmus MC
Interne Geneeskunde
Postbus 2040
3000 CA ROTTERDAM

Projectgegevens / Project information

Samenvatting / Summary

Doelstelling project:
Verbeteren van medisch specialistische zorg voor patiënten met somatisch onvoldoende verklaarde klachten (SOLK) door ontwikkelen en onderzoeken van de effectiviteit en doelmatigheid van training consultvaardigheden voor medisch specialisten gericht op patiënten met SOLK.

Vraagstelling:
1. Wat is het effect van een training consultvaardigheden voor medisch specialisten gericht op patiënten met SOLK op ongerustheid, beloop van klachten en functioneren bij patiënten met SOLK in vergelijking met ongetrainde medisch specialisten?
2. Leidt de training consultvaardigheden voor medisch specialisten gericht op patiënten met SOLK ertoe dat specialisten deze vaardigheden meer toepassen bij patiënten met SOLK in vergelijking met ongetrainde medisch specialisten?
3. Is de training consultvaardigheden kosten-effectief?

Studie opzet:
RCT met individuele randomisatie op artsniveau, interventie op artsniveau en effectmeting op patiënt- en artsniveau.

Studiepopulatie:
1. Medisch specialisten en aios van Erasmus MC.
2. Poliklinische patiënten met klachten, die de deelnemende specialist beoordeelt als SOLK.

Interventie:
Training consultvaardigheden gericht op patiënten met SOLK voor medisch specialisten.

Uitkomstmaten:

Vraag 1. Op patiëntniveau meten we met vragenlijsten: ongerustheid over klachten met Whitely Index; ernst huidige klacht met VAS schaal; distress, depressie, angst en somatisatie met 4-DKL; functioneren met SF-36.
Vraag 2. Op artsniveau meten we de toepassing van consultvaardigheden door observatie van videoconsulten en gegevens uit patiëntendossiers.
Vraag 3. Een economische evaluatie wordt uitgevoerd vanuit maatschappelijk perspectief. Als kosten worden meegenomen de kosten van interventie, gezondheidszorg (consulten, opname, thuiszorg, medicijnen), persoonlijke aard en productieverlies (absenteeism en presenteeism). Het onderzoeksteam registreert de interventiekosten, de overige volumina meten we met patiëntvragenlijsten.

Sample size en power:
Om op artsniveau een verbetering in toegepaste consultvaardigheden van 20% te kunnen aantonen met een tweezijdige alfa van 5% en een beta van 10%, zijn naar schatting 55 artsen per groep nodig. Rekening houdend met 10% uitval streven we naar het insluiten van 60 artsen per groep. Op patiëntniveau is de power dan ruim voldoende. Op de SF-36 wordt een verschil van 10% klinisch relevant geacht. Om dit aan te tonen zijn 110 patiënten per groep nodig. Wij plannen 129 patiënten per meting per groep beschikbaar te hebben voor analyse.

Tijdpad:
Maand 1-2 voorbereiding, 3-20 dataverzameling en interventie, 21-24 analyse en rapportage.

SUMMARY
Objective: To improve medical specialistic care for patients with medically unexplained physical symptoms (MUPS) by developing and researching the effectiveness and efficiency of a consultation skills training programme for medical specialists focused on MUPS-patients.

Study questions:
1.What is the effect of training medical specialists in MUPS-focused consultation skills on illness worries, course of symptoms, and daily functioning of MUPS-patients in comparison with non-trained specialists?
2.Do trained doctors use more effective communication compared to non-trained ones?
3.Is the training course cost-effective?

Design: RCT with individual randomisation and intervention on doctor-level and effect measurement on patient- and doctor-level.

Study population:
1. Medical specialists and trainees of Erasmus MC.
2. Out-patients with MUPS, diagnosed by the participating specialist.

Intervention: MUPS-focused consultation skills training course for medical specialists.

Outcome measures:
Q 1: On patient-level with questionnaires we measure: illness worries with Whitely Index; current symptom severity with VAS; distress, depression, anxiety and somatisation with 4-DSQ; daily functioning of patients with SF-36.
Q 2: On doctor-level we measure the application of consultation skills by observation of video-taped consultations and facts from patientfiles.
Q 3: An economic evaluation will be performed from societal perspective. Costs of training programme, health care utilization, personal expenses, production loss will be measured. The researchteam registers costs of intervention; we measure the other volumina by patient questionnaires.

Sample size calculation:
To detect a 20% improve in consultation skills of doctors with a 5% two-sided alpha and a 10% beta, an estimated number of 55 doctors per group will be needed. Allowing for 10% drop-out of doctors we aim to recruit 60 doctors per group. On patient level a sufficient study power will be reached. On the SF-36 outcome measure a minimal difference of 10% is considered relevant and can be detected with 110 patients per group. We plan 129 patients per group per measurement to be available for analysis.

Time-schedule:
Month 1-2 preparation, 3-20 datacollection and intervention, 21-24 analysis and report.

Trefwoorden / Keywords
medically unexplained physical symptoms; MUPS; education; communication; medical specialists; illness worries; cost-effectiveness; Randomised Controlled Trial

Samenwerking / Collaboration

Samenwerking tussen onderzoek en praktijk / Cooperation between research and practice:
Ja / Yes

Organisaties

Erasmus MC
Interne Geneeskunde
Postbus 2040
3000 CA ROTTERDAM

Erasmus Universiteit Rotterdam
Faculteit der Sociale Wetenschappen
Instituut voor Psychologie
Postbus 1738
3000 DR ROTTERDAM

Nederlands Instituut voor onderzoek van de gezondheidszorg (NIVEL)
Postbus 1568
3500BN UTRECHT

VU medisch centrum
EMGO Institute for Health and Care Research
Huisartsgeneeskunde
Van der Boechorststraat 7
1081 BT AMSTERDAM

Inhoud / Content

Probleemstelling / Problem definition

Having physical symptoms is quite normal: in population surveys 85-95% of respondents report at least one symptom during the preceding week. The decision to seek medical help is determined more by cognitions about cause, prognosis or need for medical intervention, than by severity of the symptom. In 30-50% of the physical symptoms presented in general practice the symptoms remain medically unexplained (MUPS). In hospital practice this figure is even higher: 40-60% of the presented symptoms remain medically unexplained. Although most of these symptoms disappear within a few weeks or months, still 20-30% of MUPS persist for more than a year. Patients with persistent MUPS often have reduced subjective health with impaired physical, mental or social functioning, increased prevalence of co-morbid depressive or anxiety disorders and increased sick-leave (De Waal 2004). MUPS are more prevalent among women and associated with older age, low education, employment disability or unemployment (Verhaak 2006).
Many patients with persisting MUPS have feelings of not being taken seriously by their doctors, while their doctors often feel unable to find common ground and to get agreement on a common problem definition (Salmon 2007). Patients are easily offended by incongruent messages about a supposed non-somatic origin of the symptoms, experiencing lack of empathy and acceptance for the physical symptoms. Cultural and language barriers may increase the communication problems. These doctor-patient communication problems can hamper a proper exploration of problems and an adequate treatment for persistent MUPS.

Several studies of patients' perspective on consultation showed the importance of patients seeking legitimacy for their problems (Zavestoski 2004, Peters 1998). Patients want to feel that their doctor accepts that the symptoms are real and they want doctors' attention for that. Whereas patients seek legitimacy for their physical symptoms, many doctors regard these symptoms as not warranting their help or they attribute the symptoms to psychological problems. Whereas patients want engagement, doctors have described feeling that engagement is pointless. Doctors fluctuate in their willingness to engage with patients' emotional cues depending on mood or the pressure of work (Cocksedge 2005).
Many patients with MUPS demand for doctors engagement will not be satisfied in that need. Dissatisfaction and pressure on the doctor-patient communication are often the results. Qualitative analyses have shown how doctors' explanations can create common ground that allows patients and doctors to address both psychosocial and physical problems and to avoid somatic interventions (Dowrick 2004, Salmon 1999). In a randomised, non-blinded study patients who received a firm diagnosis for their symptoms were more likely to improve than those who received no explanation (Thomas 1987). Simple explanation by the doctor or nurse has improved reassurance by normal tests (Petrie 2007).
Teaching about explanation in medical education is limited. Explanation as a skill is underestimated in the curriculum. In order to improve the care for MUPS patients the skills of explanation of MUPS and reassurance has to be developed not only by GPs but also by medical specialists.

Relevantie / Relevance

Of patients presenting physical symptoms in outpatient clinics, such as gynaecology, neurology or rheumatology, at least 40% of patients have no physical disease (Nimnuan e.a., 2001; Maiden, 2003; Reid, 2001).
These symptoms burden patients as well as health services due to large quantities of physical intervention, even surgery, which is often ineffective (Stanley, 2002; Barsky, 2005).

The health outcome of patients with MUPS can be influenced positively by patient centered communication, effective reassurance, reliable patient information and a positive explanation (Dowrick e.a., 2004; Fassaert e.a., 2008). The problem is that doctors primarily use a medical approach in their consultations with MUPS patients and reinforce unconsciously the attention of the patients towards the somatic nature of their ailments (Kappen & Van Dulmen, 2008).

To identify the basis of the communication problems that characterise consultations with MUPS patients a literature review on this subject showed that contest between patients' authority, resting on their knowledge of their symptoms and practitioners' authority, based on the normal findings of tests and investigations often is very much involved (Salmon, 2007). The outcome of consultations can be influenced by the strategies that patient and doctor use to press their authority, rather than on clinical need. Finding sensible, common ground in the explanation of the symptoms, shared by doctor and patient, is therefore very much indicated as a preferred stategy. The practitioner needs to fashion explanation that is acceptable to both parties from available medical and lay material. That means clinical education programmes should include curricula in symptom explanation focused on MUPS- patients.

The intervention under study is an effort to provide in such an education programme for medical specialist and trainees. The curriculum for medical specialists lacks the subject of consultation skills training for MUPS patients. The pilot of this training programme for neurologists in the Erasmus MC, described under 'clinical study', created a unique opportunity for the Central Education Committee to improve the quality of the interdisciplinary medical specialistic care for MUPS-patients.

The proposed study meets the criteria of the VEMI programme because the patientgroup with MUPS is at risk to become a frequent and longterm user of medical health services. In case of ailments like CFS, IBS and fybromyalgy the diagnostic proces usually takes a long time with extra unnecessary tests, which is costly for the society and a personal burden for the patient. Because medical interventions are not always available for patients with MUPS communication is even more important to maintain an adequate doctor-patient relationship and contribute to an effective medical care for MUPS patients. The new national multidisciplinary guideline on MUPS and somatoform disorders (MDR Trimbos-instituut, CBO, 2009) advises a dual policy (exploration of both the somatic aspects and psychological aspects such as the cognitions, emotions, illness worries and impact of symptoms for daily functioning of the patient), effective reassurance, positive explanation, adequate registration and report to the GP.

Effective consultation focused on MUPS-patients prevents the development of somatic disorders, improves the quality of life of MUPS-patients and the realisation of cost reduction due to the prevention of useless medical specialistic interventions.
The intervention in the proposed study contains a consultation skills training programme for medical specialists according to the above mentioned national multidisciplinary evidence based guideline on MUPS and somatoform disorders.

Kennisoverdracht, implementatie, bestendiging / Knowledge transfer, Implementation Consolidation

If the intervention under study will prove to be effective, the result will be an improvement of specialist care for patients with MUPS. This can be described in several products and new knowledge on various levels.

Target groups for implementation activities are:
1. Medical specialists in the Erasmus MC (local)
2. Medical specialists in the educational area of the Erasmus MC (South-West region of the Netherlands)
3. Academy for Medical Specialists in the Netherlands (national level)
4. Specific (inter)national organisations for medical specialists e.g.
5. Patient information providers: EDBR digital health portal (local)
6. Organisation of Dutch hospitals: SRZ (local), VNZ (national) and NFU (national academic)
7. GPs in the region and in the Netherlands (NHG).
8. General public (consisting of potential MUPS-patients)

All target groups will be advised by the end of this study.

Products: a description of the intervention i.c. the training will be available for use in other university medical centres. The training will become a part of the curriculum for medical specialists in the Erasmus MC (e.g. neurology, internal medicine, gynaecology).
For patients the information about having symptoms without a medical explanation will be made available through a professional website (in cooperation e.g. with EDBR, a digital health portal for the Rotterdam region to provide patients with information about health and diseases and more specific information for reassuring).
Knowledge: The results on effectiveness of trained medical specialists will be disseminated by two international and one Dutch publication. The outcomes on effectiveness and efficiency will be made availabe for healthcare insuring companies to develop insuring policies about payment of enhanced medical specialistic care for MUPS-patients.
Practical knowledge:the training consultation skills for medical specialists focused on MUPS-patients will be part of the educational programme of The Academy for Medical Specialists.
Implementation will be facilitated by the active role of several project members in key-positions for training medical specialistst. The outcomes and experiences will be shared with the public by newspaper articles.

Doelstelling / Objective

The aim of the study is: To improve specialistic care for patients with MUPS by development and research of the effectiveness and efficiency of a consultation skills training programme for medical specialists focused on patients with MUPS.

The primary study question is:
1. What is the effect of training medical specialists in MUPS-focused consultation skills on illness worries, course of symptoms and daily functioning of MUPS-patients in comparison with non-trained specialists?

Secundairy study questions are:
2. Do trained medical specialists use more effective communication compared to non-trained ones?
3. Is the training course cost-effective?

Plan van Aanpak / Strategy

CLINICAL STUDY
Preliminary studies by applicants:

Blankenstein performed a cluster-randomised RCT with 162 somatising patients on effectiveness of cognitive-behavioural techniques applied by trained GP to patients with MUPS. In a 20-hour GPtraining programme, intervention GPs learned to apply reattribution combined with a cognitive-behavioural approach to persistent illness worries. This appeared to be a feasible intervention that could be applied during normal surgery hours. In the RCT the intervention was compared to usual GP care. From baseline to two-year follow-up, subjective health increased in the intervention group from 36/100 to 74/100, and from 36/100 to 39/100 in the control group (p<.001). Total number of health care visits during the preceding 6 months diminished in the intervention group from 10 to 5, and remained stable in the control group: from 12 to 13 (p.017). Sick leave in weeks during the preceding 6 months decreased in the intervention group from 5 to zero, and remained stable in the control group: from 4 to 4 (p <.001). Use of medication did not change in either of the groups. (Blankenstein, 2002)
Blankenstein has also contributed to a community survey on impact of physical symptoms on perceived health with 2447 responders (response 53%). Fatigue was the most commonly reported symptom with a prevalence of 57%, followed by headache (40%) and low back pain (39%). More than half of responders reported three symptoms or more. Increasing number of symptoms is strongly associated with poorer physical, emotional and social functioning. (Van der Windt 2008).
Blankenstein is currently performing a Cochrane systematic review on psychiatric consultation letters for MUPS. (Hoedeman, Blankenstein 2007). Blankenstein performed a study on determants of successful patient recruitment in research [van der Wouden, Blankenstein et al. 2007]

Apart from this the project advisor on this proposed study found that consultations for medically unexplained physical symptoms (MUPS) constitute a large part of the workload of a broad scale of medical specialists, i.e. internists, gastroenterologists, gynaecologists, pediatricians, etc. As medical interventions are not always available, specific communication strategies might help physicians to handle these problems adequately. Although such a strategy seems quite logical, recent research of the NIVEL communication group examining ninety-seven videotaped medical visits from patients presenting MUPS shows that physicians primarily use a medical approach in their communication (Kappen & Van Dulmen, 2008). Such an approach might incorrectly confirm the somatic nature of the patient’s condition. As results also show that most patients present their concerns only in an implicit way, physicians may need to be taught to recognize and explore patients’ concerns more actively. Findings from another NIVEL study indicate that such an approach might indeed be successful with MUPS-patients. In this study, the use of positive communication strategies during 524 videotaped consultations with patients with minor ailments was analyzed and related to the visit outcome in terms of medication adherence, consultation frequency, functional health status and state anxiety. Results show that, to some extent, it seems helpful when GPs are at the same time clear and optimistic about the nature and course of minor ailments. Although communication strategies might to some extent contribute to the management of minor ailments, the results of this observational study also indicate that it is important for a physician to pay attention to the mood of the patient who enters the consulting room (Fassaert et al, 2008). Previous research with 120 patients with IBS visiting an internist indicates that for this purpose a minimal psychological intervention provided by a physician can already be effective in ameliorating MUPS symptoms (Van Dulmen et al, 1994; 1996; 1997). These results will be included in the training which will be offered to the medical specialists in the proposed study.

Weiland performed a pilot of the intervention ' consultation skills training programme for medical specialists focused on MUPS-patients' in cooperation with the Department of Neurology Erasmus MC Rotterdam (2007, 2008). In two different groups a total of 22 neurologists (5 staff members and 17 medical specialist trainees) participated in this training with an attendency rate of 90%.
In 4 training sessions with an interval of 4 to 6 weeks the neurologists and trainees learnt consultation skills focused on MUPS-patients, which they practised during consulting hours in between the sessions. The training model was based on learning-by-doing and had the character of workshops, in which step by step new skills were introduced, practised and supervised. In small groups of three participants role-plays were the educational instrument (doctor-patient-observer). The use of individual case-material was enhanced.

In session 1 the basics of MUPS focused consultation skills were taught:
- Exploring of patients complaints following the SCEBS-model (SCEBS: Somatic symptoms, Cognitions, Emotions, Behaviour and Social environment).
- Informing the patient in positive vocabulaire, with referral to the patients cognitions/emotions and with clear explanations such as “the lungs are clean and I hear a healthy sound of your breath”. Special attention was given to vicious circles and plausible explanations.
- Planning and making appointments with patients in a SMART way: little steps ahead which can be accomplished successfully.

In session 2 gathering more information of the patient by registration of its own complaints and searching for new connections together with the patient was practised:
- Purpose of the registration
- Motivating patients
- Giving clear instructions
- Design of the registration
- Planning and discussing with the patient.
- Reattribution: maximization of patients reception to a new understanding of his complaints and acceptance of other explanations of the symptoms than ‘it must be a disease’.

In session 3 skills for patients with persistent illness worries were introduced and report to the referring doctor was discussed:
- The art of reassuring patients
- Questioning patients about their dramatic thoughts
- Discussing the impact of these scenarios
- Putting these thoughts to the test (reality-check)
- Evaluation: change in patient perception of complaints
- Managing expectations about possible testresults
- Addressing health anxiety
- Dealing with referral letters
- Report to GP

In session 4 the participants presented their experiences with the new knowledge and skills:
- Review of treatment of a patient with the accomplished skills in couples
- Discussion and feedback
- Practising skills when necessary

The pilot was evaluated by a questionnaire (N=19). Results of the evaluation in terms of useful (not, medium or very useful) show:
* Recommendation of the training model (practising-step-by-step and learning-by-doing) due to supervision of trainers and feedback of participants.
* Exploring 'SCEBS' as the most useful experienced skill because its impact on control for the doctor in the communication with (all kind of) patients (by 18 participants).
* Practising of reassuring patients effectively was evaluated as very useful (by 16 participants).
* Informing patients about possible interrelating factors reinforcing their complaints was as skill equally evaluated with the management of expectations (14 participants said very useful).
* The participants evaluated the skills ‘planning SMART activities with patient’ and ‘reattribution’ as medium useful (by 11 participants) due to logistical problems in the follow-up of out-patients.
* Adjusting the reader for medical specialists was evaluated as an improvement for the course.
* Guided intervision as a follow-up of the training was mentioned as a need for permanent education on this subject.

Results were presented to the Head of Department of Neurology and the Central Education Committee Erasmus MC. The pilot-training was discussed in the research team and adjustments for improvement of the intervention have been made for this proposed study.

DESIGN:
RCT with randomisation and intervention on doctor-level and effect measurement on patient- and doctor-level.
Setting: Out-patient clinic of the Erasmus MC.
Study population: medical specialists and trainees within Erasmus MC and their out-patients with MUPS.

INTERVENTION:
Training consultation skills for medical specialists focused on patients with MUPS.

The specialists receive 4 training sessions, with an interval of 4 to 6 weeks, in groups of 12 persons with 2 experienced trainers (trainers receive special training and supervision); 5 intervention- and 5 control groups will be composed. After individual randomisation of doctors 10 training groups will be composed in a way that doctors of different medical specialisms are mixed together in a group, in order to stimulate the broader scope, which is necessary for patients with MUPS.

The medical specialists will be taught:
1- to notice and respond to the concerns of the patient
2- to register these concerns in the (electronic) patient record
3- to address the reasons for referral
4- to reassure the patient
5- to give a positive explanation, in which the doctor is emphasizing that and how symptoms frequently occur without medical diagnosis or disease.
6- to speak clearly and motivational with the patient
7- to write a letter to the patient's GP in which the explanation given to the patient is resumed, and recommendations on policy and lifestyle are reported.

For the items 1, 4, 5 and 6 the evidence based course of the Dutch College of General Practitioners ‘GP-policy for medically unexplained physical symptoms’ can be used with slight adjustments, for items 2, 3 and 7 new education will be developed.

Doctors in the control group receive the training consultation skills afterwards, i.c. when the RCT is finished.

Recruitment of doctors:
Medical specialists and trainees of the participating medical specialisms are included in the research on the premises that (1) they are prepared to participate in the training and the measurements before and after the training; (2) they agree that their MUPS-patients will be asked to participate in the research; (3) they perform consultations with out-patients during the research period.

Recruitment of patients:
Doctors are video-taping consultations with an unmanned camera during the out-patient clinic with informed consent of the patient. After every consultation the doctor will determine whether the physical symptoms of the patient can be unsufficiently explained by a medical disease (= MUPS, most likely 30-50%). These MUPS-patients will be asked to participate in the research (pre-training group). After training again doctors will video-tape consultations and patients again will be asked to participate in the research (post-training group). The patients in the post-training group are not the same nor overlapping with patients in the pre-training group.
A total number of 720 MUPS-patients will be video-taped and, when informed consent is given by these patients, analysed. With a response rate of 80% the questionnaires of 576 patients will be analysed.
To avoid failing patient recruitment by busy doctors, students will be recruited to organise the recording of video-tapes, and to ask informed consent from eligible patients.
The inclusion period consists of 5 rounds: during 5 months every month 24 specialists and trainees with MUPS-patients will be included.

Randomisation: at the end of every inclusion round, after recording of video consults and inclusion of pre-measurement patients, the 24 doctors of that round will be individually randomised and stratified for medical discipline and medical specialists versus vocational trainees. Assigning by lot, by an independent person, 12 doctors with their MUPS-patients will be allocated to an intervention- and 12 to a control group.

FEASIBILITY OF DOCTOR RECRUITMENT:
Based on the outcome of an inquiry by questionnaire in the Erasmus MC the feasibility of doctor recruitment is sufficient. Nine medical specialisms are willing to schedule the training 'consultation skills for medical specialists focused on MUPS patients' for their staff and trainees (total number of available participants is 134 trainees and 18 medical specialists; for the trainees the training will be part of the interdisciplinary curriculum). Participation will be stimulated actively by prof. dr. J.L.C.M. van Saase, head of the Internal Medicine education programme and Chair of the Central Education Committee Erasmus MC.
Feasibility of the time-schedule is also sufficient: intervention already is almost developed, data collection can start quickly and being accomplished within 15 months. Analysis and report take place while control groups receive their training. Sufficient supporting research assistants are a necessity to perform the project in 2 years in the Erasmus MC.

OUTCOME PARAMETERS:
Research question 1. On patient-level we will measure: illness worries of patients about their complaints with the Whitely Index; symtom severity with VAS scale(s) for the current symptom(s; distress, anxiety, depressive symptoms and somatisation with the 4-DSQ [Terluin]; impact of symptoms on daily functioning with the SF-36.
Research question 2. On doctor-level we will measure the practicing of consultation skills in real-patient consultations with MUPS patients: trained observers will score items 1, 3, 4, 5 and 6 by observation of video-taped consultations. A standardized observation scoring list will be developed, based on the Roter Interaction Assessment System (RIAS), containing only those aspects relevant for measuring item 1, 3, 4 and 5; items 2 and 7 will be extracted from the patient records. To enhance the reliability of the consultation skills assessment, before the training 3 consultations with MUPS patients and after the training another 3 consultations will be video-taped and assessed for every doctor. Fifty videotaped consultations will be double observed and scored to assess the interrater reliability.
Research question 3. An economic evaluation will be performed from societal perspective. As costs will be involved the direct costs of the intervention (training course + time-investment of medical specialists), costs of healthcare (number of attendings to GP, medical specialists, Mental Health Care, paramedical care, alternative healers; hospital admission days; hours homecare; prescribed medicines), costs for patient and family (transportation costs, hours family care, self medication), and indirect costs due to loss of production (absenteeism and presenteeism). The research team registers the intervention costs, the other volumina will be measured with patient questionnaires.

Measurement timings and - procedures:
In the month after inclusion of the doctors pre-measurement consultations will be videotaped and the patients with MUPS in the pre-training group will be included. These patients will be asked to answer the questionnaire directly after the consultation and after 3 and 6 months. In the meantime the doctors in the intervention group receive the training.
Six months after the inclusion of the doctor the post-training consultations will be videotaped and the patients with MUPS in the post-training group will be included. These patients also will be asked to answer the questionnaire immediately after the consultation and after 3 and 6 months. For logistic reasons students will be involved during the process of data collection.

Statistical analysis:

Research question 1. Differences between intervention- and control patients (clustered within doctors) will be measured with the WI, VAS, 4-DSQ and SF-36 at 3 and 6 months in the pretraining- and posttraining groups; differences will be addressed at baseline with multilevel analysis on doctor- and patient-level (SAS Proc Mixed).
Research question 2. Separate scores on the 7 items each as well as a sum score on consultation skills will be analyzed at doctor-level. Therefore item- and total scores of 3 consultations (patients) in the pretraining group and 3 consultations (patients) in the posttraining group of the same doctor will be compared. Differences in the posttraining scores between intervention- and control doctors will be adjusted for their pretraining scores, and will be analyzed with the General Linear Mixed Model (SAS Proc Mixed). Contrast analyses will be planned for the demographic characteristics of the patients, whether it is a first versus a follow-up consultation of the patient, and (senior) specialists versus trainees.
Research question 3. If available, costs will be calculated using standard prices (Oostenbrink list). Care by family members will be calculated by using shadow prices. Total costs per patient will be estimated on the basis of direct as well as indirect costs. Analysis will include bootstrapping techniques. Results will be presented as incremental cost effectiveness ratios, of which reliability will be presented in cost effectiveness diagrams.

SAMPLE SIZE CALCULATION:
To detect a 20% improvement in consultation skills of doctors with a 5% two-sided alpha and a 10% beta, an estimated number of 55 doctors per group will be needed. Allowing for 10% drop-out of doctors we aim to recruit 60 doctors per group. Because we have three pretraining patients and three posttraining patients per doctor, on patient level a sufficient study power will be reached easily. On the SF-36 outcome measure a minimal difference of 10% is generally considered as clinically relevant. This difference can be detected with 110 patients per group. We aim at 129 patients per group, both for pre- and for post-measurement available for analysis.

ECONOMICAL EVALUATION
-General considerations
To judge the training in terms of its cost-effectiveness,
where effects on 'quality of life' are expressed in terms of money, we will perform cost-effectiveness analysis. Besides, we expect reduced costs for medical treatment in the intervention group when we compare the posttraining group to the pretraining group. To see whether the benefits of reducing the direct and indirect costs outweigh the costs of the training, both in terms of money, we will also perform a cost-benefit analysis.
-Cost analysis
We will calculate the costs of the burden of disease of MUPS for the patients in this project with a questionnaire on direct and indirect costs associated with MUPS measured over last month. Direct costs of the intervention are e.g. the costs of the trainingscourse, the time-investment of medical specialists and of the trainer. Other direct costs concern the costs of resources for medical treatment including prescribed medicines and alternative healers.
Finally we will measure costs for patient and family (transportation costs, hours family care, self medication), and indirect costs due to loss of production (absenteeism and presenteeism). The research team registers the intervention costs, the other volumina will be measured with patient questionnaires per month.
-Patient outcome analysis
To measure the effects of the training we will use following instruments: the WI, VAS, 4-DSQ and SF-36 at baseline, after 3 months and after 6 months in the pretraining group (intervention- as well as control group) as well as the posttraining group (intervention- as well as control group).

SYSTEMATIC REVIEW:
In the intervention of the proposed study, a consultation skills training programme for medical specialists focused on MUPS-patients, the ability to reassure patients effectively is one of the most important skills to improve. In the Dutch multi-disciplinary guideline for MUPS (Trimbos-institute in co-operation with CBO, 2009) one of the applicants (Blankenstein) performed as committee-member/editor the systematic review on MUPS and reassurance.
The study question was: ‘What is the evidence for the impact of reassurance in MUPS patients on the prevention of somatoform disorders’?
Results: four relevant studies were identified. In a qualitative study of interpretation of reassurance among patients attending rheumatology clinics (Donovan & Blake, 2000) 35 patients were interviewed before and after visiting the medical specialist. Analysis of the recorded interviews showed that reassurance played an important role in the information given by the specialist. The doctors were emphasising the early stage or the mildness of the disease and the chance on recovery. This information was not reassuring patients, but even strengthened patients' alarming cognitions about pain and invalidity. Patients who noticed that their doctor was addressing their problems felt more reassured.
The conclusion of this and other studies (Dowrick, 2004; Ring, 2004; Epstein 2007) is there are indications that doctors use often inadequate skills for reassuring, such as explanations which are not fitting the patient, medical actions and treat the symptoms of little importance.

A broad range of MeSH terms on MUPS, doctor-patient communication, normal test result, reassurance and psychoeducation has been performed and can be delivered on request.

TIME SCHEDULE:
Month 1-2 Preparation phase:
- research protocol, questionnaires, information materials;

- finalising training syllabus, design, educational materials
- informational mailing to doctors
- informational conference for doctors, recruiting doctors

Month 3-20 Data collection and intervention phase:
- inclusion of doctors for intervention and control group
- inclusion of MUPS patients
- randomisation of individual doctors into intervention and control condition
- pre-training group measurements by video-taping consultations and by patient questionnaires
- training intervention doctors
- post-training group measurements by video-taping consultations and by patient questionnaires
- collection of video-tapes and questionnaires
- assessment of video-taped consultations
- data-entry
- data-cleaning

Month 21-24 Analysis and report phase:
- data-analysis
- writing articles on effectiveness and economic evaluation
- final report ZonMw

Time schedule is based on inclusion scheme and flow chart.

Expertise, voorgaande activiteiten en producten / Expertise, prior activities and products

- Research on MUPS and doctor-patient communication:

Blankenstein has expertise on performing RCTs concerning doctor-patient communication: she performed a PhD study with a RCT on effectiveness of reattribution (a cognitive intervention) for somatisation in general practice, she is currently project leader of controlled trials on GP-patient communication in palliative care, patient feedback on consultations skills of GP trainees, and effectiveness of stepped care for MUPS-patients. Van Dulmen obtained her PhD degree in 1996 with the thesis titled ‘Exploring cognitions in irritable bowel syndrome: implications for the role of the doctor’. As a research coordinator of the NIVEL program Communication in healthcare Van Dulmen has gathered, as project advisor on the proposed study, a lot of expertise in the field of the communication and information process in healthcare. The investigations involve a broad range of patient groups including patients with medically unexplained physical symptoms, also the topic of the PhD thesis of Van Dulmen. A common feature of these NIVEL studies - which is also relevant for the proposed study - is the focus on real patient encounters which are video-recorded and subsequently analysed in detail using research-specific observation instruments, such as the Roter Interaction Analysis System (RIAS).

- Research on patient recruitment strategies:

Blankenstein has been project leader of the ZonMw research project 'Lasagna's law', an empirical study on determinants of failing and successful patient recruitment for reseach on general practice.

- Expertise on training programmes on MUPS for doctors:

Blankenstein has developed and supervised GP training programmes on cognitive behavioral techniques for MUPS that have been implemented in GP vocational training and post-graduate training programmes. Blankenstein and Weiland have developed modified versions for medical specialists (neurologists, psychiatrists, occupational health physicians) and both are experienced trainers in this subject. Weiland studied Adult Education at the University of Groningen (NL)and has a broad experience in the education of medical professionals. As behavioural scientist and practitioner she developed and performed consultation skills training programmes for the Departments of General Practice, Medical Psychology and Psychotherapy at the Erasmus MC and the Dutch Institute for General Practitioners (NHG). Weiland developed and performed several projects with University Hospitals and the Dutch/European Neuro Muscular Dystrophy Association aimed at the improvement of the quality of life for NMA patients. As head of the Department of Psychosocial Care in the Erasmus MC she performed a pilot consultation skills training programme focused on MUPS patients on behalf of the Department of Neurology.

- Research on effectiveness of skills training programme:

Van der Molen has expertise in the field of the development, execution and evaluation of professional communication skills training, and the development of modern instruments to measure the effects of these training programmes. He is (co-)author of about 30 books and about 150 articles and chapters, many of which concern the effectiveness of training programs.

- Research on efficiency of a medical intervention:

Arends has statistical expertise in the development of new statistical techniques for (multivariate) meta-analysis. Besides she

has ample experience in statistical consultancy, resulting in more than 30 international publications in medical journals. She is involved as Biostatistician in the Generation R Study, a cohort study of 10.000 babies in Rotterdam. Arends assists about 25 PhD students with their statistical analyses of data of the Generation R Study. When she was employed at the Trimbos Institute, Utrecht, she was one of the first researchers who were involved with cost-effectiveness analyses of the mental health care.

- Feasibility of doctor recruitment:

Van Saase is head of the Internal Medicine education program and responsible for the training of over 100 internists in the Rotterdam region. He is Chair of the Central Education Committee of the Erasmus MC. He is extraordinary professor with special attention to competence oriented education of specialists. Kuipers is professor of Internal Medicine and chair of the Department of Gastroenterology and Hepatology of the Erasmus University Medical Center. Since 2006 he also chairs the Department of Internal Medicine of the Erasmus MC. He is a member of the Dutch National Health Council, and President-elect of the Dutch Society of Gastroenterologists and the European Helicobacter Study Group. In 2006, he was awarded with a fellowship of the American Gastroenterology Association. His research focuses on the etiology, diagnosis and prevention of early neoplastic lesions of the gastrointestinal tract, and he chairs the Rotterdam Colon Cancer Screening group.

Publicaties / Publications

Arends LR & van Gageldonk A (1997). Het effect van interenties bij stemmings-stoornissen. Overzicht van meta-analyses en reviews,Trimbos-instituut, Utrecht.

Arends LR, Hamza TH, Van Houwelingen JC, Heijenbrok-Kal MH, Hunink,MG & Stijnen T (2008). Bivariate Random Effects Meta-Analysis of ROC Curves. Medical Decision Making, 28(5), 621-638.

Arends, LR, Hunink, MG & Stijnen, T. (2008). Meta-analysis of summary survival curve data. Statistics in Medicine, 27(22),4381-4396.

Blankenstein AH, van der Horst HE, Schilte AF, de Vries D, Zaat JOM, Knottnerus JA, van Eijk JTM, de Haan M. Development and feasibility of a modified reattribution model for somatising patients, applied by their own general practitioners. Patient Education and Counseling 2002;47:229-35.

Boot CR, Meijman FJ, Dulmen S van. Lay knowledge about the causes of complaints and illness; a study in primary care. Health Communication 2009;24:346-350.

Donker M, Arends L, Gageldonk A van, Roijen L van, Smit F, Bijl R.(1996). Prioriteiten voor kosten-effectiviteitsonderzoek in de GGZ, NcGv-reeks 96-15, Utrecht.

Dulmen AM van, Fennis JFM, Mokkink HGA, Velden HGM van der, Bleijenberg G. Doctors' perception of patients' cognitions and complaints in irritable bowel syndrome at an out-patient clinic. J Psychosom Res 1994;38:581-590.

Dulmen AM van, Fennis JFM, Mokkink HGA, Velden HGM van der, Bleijenberg G. Doctor-dependent changes in complaint-related cognitions and anxiety during medical consultations in functional abdominal complaints. Psychol Med 1995;25:1011-1018.

Dulmen AM van, Fennis JFM, Mokkink HGA, Velden HGM van der, Bleijenberg G. Persisting improvement in complaint-related cognitions initiated during medical consultations in functional abdominal complaints. Psychol Med 1997;27: 725-729.

Dulmen AM van, Fennis JFM, Bleijenberg G. Towards effective reassurance in irritable bowel syndrome; the importance of attending to patients' complaint-related cognitions. Psychol Hlth & Med 1998;3:405-416.

Dulmen AM van. Communication during gynaecological outpatient consultations. J Psychosom Obstet Gynaecol 1999; 20:119-126.

Dulmen AM van, Holl RA. Effects of continuing pediatric education in interpersonal communication skills. Eur J Pediatr 2000;159:489-495.

Dulmen AM van, Weert JCM van. Effects of gynaecological education on interpersonal communication skills. Br J Obstet Gynaecol 2001;108:485-491.

Dulmen S van, Nübling M, Langewitz W. Doctors’ responses to patients’ concerns; an exploration of communication sequences in gynaecology. Epidemiologia e Psichiatria Sociale 2003;12:98-102.

Dulmen S van, Sluijs E, Dijk L van, Ridder D de, Heerdink R, Bensing J. Patient Adherence to medical treatment: a review of reviews. BMC Health Services Research. BMC Health Services Research 2007, 7:55.

Dulmen S van, Tromp F, Grosfeld F, Cate Th J ten, Bensing JM. The impact of assessing simulated bad news consultations on medical students’ stress response and communication performance. Psychoneuroendocrinology 2007; 32:943–950.

Essers G, Floor E, Blankenstein AH, van der Horst HE, Romeijnders ACM. Beleid bij patiënten met lichamelijk onverklaarde klachten. Cognitief-gedragstherapeutische technieken door de huisarts. NHG 2003.

Fassaert Th, Dulmen S van, Schellevis F, Bensing J. Attentive listening in medical consultations; development of the Attentive Listening Observation Scale (ALOS-global). Patient Educ Couns 2007;68:258-264.

Fassaert Th, Dulmen S van, Schellevis F, Jagt L van der, Bensing J. Raising positive expectations helps patients with minor ailments. BMC Family Practice 2008 Jun 30;9(1):38.

Hoedeman R, Blankenstein AH, van der Feltz-Corneliz CM, Krol B, Groothoff JJW. Consultation letters for medically unexplained physical symptoms (Cochrane protocol). In: The Cochrane Library,Issue 2,2007.Chichester,UK: John Wiley & Sons,Ltd.

Kappen T, Dulmen S van. General practitioners' responses to the initial presentation of medically unexplained symptoms: a quantitative analysis. BioPsychoSocial Med 2008, 2:22.

Kuntze J, Van der Molen HT & Born MPh (2009). Increase in Counseling Communication Skills after Basic and Advanced Microskills Training. British Journal of Educational Psychology,79,175-188.

Lang G, Molen HT van der, Trower P, Look R (1990). Personal Conversations. Roles and Skills for Counsellors. London: Routledge.

Liem, Y.S., Bosch, J.L., Arends, L.R., Heijenbrok-Kal, M.H. & Hunink, M.G. (2007). Quality of life assessed with the medical outcomes study short form 36-item health survey of patients on renal replacement therapy: A systematic review and meta-analysis. Value in Health, 10(5), 390-397.

Molen HT van der, Klaver A, & Duyx M (2004). Effectiveness of a communication skills training program for the management of dental anxiety. British Dental Journal,196,101-108.

Molen HT van der, Nijenhuis J te & Keen G (1995). The Effects of Intelligence Test Preparation. European Journal of Personality,9,p.43-56.

Molen HT van der, Gramsbergen YH (2005). Communication in Organizations. Basic Skills and Conversation Models. London: Routledge.

Morren M, Dulmen S van, Ouwerkerk J, Bensing J. Compliance with momentary pain measurement using electronic diaries: A systematic review. Eur J Pain 2009; 13:354-365.

Qureshi NA, Molen HT van der, Schmidt HG, Al-Habeeb TA, Magzoub MEM (2006). Effectiveness of a Training Programme for Primary Care Physicians Directed at the Enhancement of their Psychiatric Knowledge in Saudi Arabia. Education for Health,19,52-60.

Reinders ME, Blankenstein AH, van Marwijk HWJ, Schleypen H, Schoonheim PL, Stalman WAB. Development and feasibility of a patient feedback programme to improve consultation skills in general practice training. Patient Education and Counseling 2008;72:12-9.

Saase JLCM, et al. Opleidingsplan Interne Geneeskunde 2009; ISBN 978-90-8523-2018.

Saase JLCM, et al. The future medical specialist. NFU publication. In press.

Schmidt HG, Vermeulen L, Molen HT van der (2006). Longterm effects of problem-based learning: a comparison of competencies acquired by graduates of a problem-based and a conventional medical school. Medical Education,40,562-567.

Schönrock-Adema J, Van der Molen HT, Van Oudenhoven-Van der Zee KI (2009). Effectiveness of a Self-Instruction Program for Microcounseling Skills Training. Teaching of Psychology,36,246-252.

Van der Windt DA, Dunn KM, Spies-Dorgelo MN, Mallen CD, Blankenstein AH, Stalman WA. Impact of physical symptoms on perceived health in the community. Journal of Psychosomatic Research 2008;64(3):265-74.

Van der Wouden JC, Blankenstein AH, Huibers MJH, Van der Windt DAWM, Stalman WAB, Verhagen AP. Survey among 78 studies showed that Lasagna's law holds in Dutch primary care research. Journal of Clinical Epidemiology 2007; 60(8):819-824.

Van Houwelingen HC, Arends LR & Stijnen T (2002). Advanced methods in meta-analysis: multivariate approach and meta-regression. Statistics in Medicine,21(4),589-624.

Van Roijen L & Arends LR (1996). Kosten van psychische ziekten, iMTA/NcGv, Rotterdam.

Visser, M.J., Postma, D.S., Arends, L.R., de Vries, T.W., Duiverman, E.J. & Brand, P.L. (2001). One-year treatment with

different dosing schedules of fluticasone propionate in childhood asthma - Effects on hyperresponsiveness, lung function, and height. American Journal of Respiratory and Critical Care Medicine, 164 (11), 2073-2077.

Referenties / References

Barsky AJ, Orav EJ, Bates DW. Somatization increases medical utilization and costs independent of psychiatric and medical comorbidity. Arch Gen Psychiat 2005;62:903-10.

Cocksedge S, May C. The listening loop: a modelof choice about cues within primary care consultations. Med Educ 2005;39:999-1005.

Donovan JL, Blake DR. Qualitative study of interpretation of reassurance among patients attending rheumatology clinics:'jst a touch of arthritis, doctor?', 2000, Britisch Medical J; 320: 541-544.

Dowrick CF, Ring A, Humphris M, Salmon P. Normalisation of undexplained symptoms by general practitioners: a functional typology. British Journal of General Practice 2004;54:165-70.

Epstein RM, Hadee T, Carroll J, e.a. 'Could this be something serious?'. Reassurance, uncertainty and empathy in respons to patients'expressions of worry. Journal of General Internal Medicine 2007; 22: 1731-1739.

Howard L, Wessely S, Leese M, e.a. Are investigations anxiolytic or anxiogenic? A randomised controlled trial of neuroimaging to provide reassurance in chronic daily headache. Journal of Neurology, Neurosurgery and Psychiatry, 2005;76:1558-1564.

Maiden NL, Hurst NP, Lochhead A, Carson AJ, Sharpe M. Medically unexplained symptoms in patients referred to a specialist rheumatology service: prevalence and associations. Rheumatology (Oxford) 2003;42:108-12.

Multidisciplinaire Richtlijn Somatisch Onvoldoende verklaarde Lichamelijke Klachten en Somatoforme Stoornissen, 2009. Trimbos-instituut en het Kwaliteitsinstituut voor de Gezondheidszorg CBO in opdracht van de Landelijke Stuurgroep Multidisciplinaire Richtlijnontwikkeling in de GGZ.

Nimnuan C, Hotopf M,Wessely S. Medically unexplained symptoms: an epidemiological study in seven specialities. J Psychosom Res 2001;51:361-7.

Peters S, Stanley I, Rose M, Salmon P. Patients with medically unexplained symptoms: Sources of patients'authority and implications for demands on medical care. Soc Sci Med 1998;46:559-65.

Petrie KJ, Muller JT, Schirmbeck F, Donkin L, Broadbent E, Ellis CJ, Gamble G, Rief W. Effect of providing information about normal test results on patients reassurance: randomised controlled trial. Brit Med J 2007;334:352.

Reid S, Wessely S, Crayford T, Hotopf M. Medically unexplained symptoms in frequent attenders of secondary health care: retrospective cohort study. Brit Med J 2001;322:767

Ring A, Dowrick C, Humphris G, e.a. Do patients with unexplained physical symptoms pressurise general practitioners for somatic treatment? A qualitative study. 2004. Britisch Medical Journal, 328: 1057.

Salmon P, Peters S, Stanley I. Patients perception of medical explanations for somatisation disorders: qualitative analysis. Brit Med J 1999;318:372-6.

Salmon P, Dowrick CF, Ring A, Humphris GM. Voiced but unheard agenda’s: qualitative analysis of the psychosocial cues that patients with unexplained symptoms present to general practitioners. British Journal of General Practice 2004;54:171-6.

Salmon P. Conflict, collusion or collaboration in consultations about medically unexplained symptoms: The need for a curriculum of medical explanation. A review. Patient Education and Counseling 2007;67:246-254.

Speckens AEM, Van Hemert AM, Spinhoven Ph, and Bolk JH. The diagnostic and prognostic significance of the Whitely Index, the Illness Attitude Scales and the Somatosensory Amplifcation Scale. Psychol Med 1996; 26: 1085-1090.

Stanley IM, Peters S, Salmon P. A primary care perspective on prevailing assumptions about persistent medically unexplained physical symptoms. Int J Psychiat Med 2002;32:125-40.

Terluin B. De Vierdimensionale Klachtenlijst (4DKL). Een vragenlijst voor het meten van distress, depressie, angst en somatisatie. Huisarts Wet. 1996;39:538-547.

Terluin B, van Marwijk HW, Adèr HJ, de Vet HC, Penninx BW, Hermens ML, van Boeijen CA, van Balkom AJ, van der Klink JJ,Stalman WA. The Four-Dimensional Symptom Questionnaire (4DSQ): a validation study of a multidimensional self-reportquestionnaire to assess distress, depression, anxiety and somatization. BMC Psychiatry. 2006;6:34.

Thomas KB. General-practice consultations - Is there any point in being positive? Brit MEd J 1987;294:1200-2.

Verhaak PFM, Meijer SA, Visser AP and Wolters G. Persistent presentation of medically unexplained symptoms in general

practice. Family Practice 2006; 23: 414-20.

De Waal MWM, Arnold IA, Eekhof JAH, et al. Somatoform disorders in general practice: prevalence, functional impairment, and comorbidity with anxiety and depressive disorders. British Journal of Psychiatry 2004;184:470-6.

Ware JE Jr. SF-36 health survey update. Spine. 2000;25(24):3130-9.

Zavestoski S, Brown P, McCormick S, Mayer B, D'Ottavi M, Lucove JC. Patient activism and the struggle for diagnosis: Gulf War illnesses and other medically unexplained physical symptoms in the US. Soc Sci MEd 2004;58:161-75

Financiële gegevens / Financial data

ZonMw budget

	**Jaar / Year**								
**Kostenpost**	*1*	*2*	*3*	*4*	*5*	*6*	*7*	*8*	**Totaal / Total**
Personeel	53,538	57,500	0	0	0	0	0	0	111,038
Materieel	7,000	5,000	0	0	0	0	0	0	12,000
Implementatie	0	0	0	0	0	0	0	0	0
Apparatuur	0	0	0	0	0	0	0	0	0
Overig	0	0	0	0	0	0	0	0	0
**Totaal / Total**	**60,538**	**62,500**	**0**	**0**	**0**	**0**	**0**	**0**	**123,038**

Co-financiering / Cofinancing

Naam co-financier / Name of cofinancier	Bedrag / Amount	Status
CZ Actief in Gezondheid	55	Wordt aangevraagd
Fonds NutsOhra	25	Wordt aangevraagd

Bijzondere gegevens / Additional information

Vergunningen / Permits

	Vergunning nodig / Permit required?		Vergunning verkregen / Permit obtained?	
	Ja / Yes	Nee / No	Ja / Yes	Nee / No
METC	X			X
DEC		X		X
WBO		X		X

Onderschrijvingen / Assents

	Ja / Yes	Nee / No
Code biosecurity / Code Biosecurity		X
Code openheid dierproeven / Code Transparency of Animal Testing		X

Andere vergunningen / Other permits

Ondertekening / Signatures

Naam projectleider en penvoerder:<br>J.L.C.M. van Saase	Naam bestuurlijk verantwoordelijke:<br>E.J. Kuipers
Plaats en datum:	Plaats en datum:

Handtekening:	Handtekening:

wetenschappelijk onderzoek

EMGO-Instituut
van der Boechorststraat 7
1081 BT Amsterdam

Postbus 7057
1007 MB Amsterdam

telefoon 020 444 8199
fax 020 444 8361

secretariaat:
hag.emgo@vumc.nl

Aan ZonMW
T.a.v. Dr. Karen van Liere-Visser
Programmasecretaris VEMI
Programma Doelmatigheidsonderzoek
Projectnummer 80-82305-97-11038

VU medisch centrum

1 januari 2010 --- 020 444 8361 --

--- 020 444 8198 ah.blankenstein@vumc.nl

Betreft Bevestiging deelname onderzoek

Geachte mevrouw Van Liere,

Het VUmc, afdeling Huisartsgeneeskunde/ EMGO+ instituut vindt het van belang om samen met het Erasmus MC onderzoek te doen naar de "Effectiviteit en doelmatigheid van een training consultvaardigheden voor medisch specialisten gericht op patiënten met somatisch onvoldoende verklaarde klachten (SOLK)".

Ondergetekende ondersteunt de uitgewerkte subsidieaanvraag en participeert actief als begeleider in dit onderzoek voor wat betreft de inhoudelijke thematiek van onderzoek op het gebied van SOLK en trainingen.

Met hartelijke groet,

Dr. A. H. Blankenstein

Afdeling Huisartsgeneeskunde, EMGO+-Instituut, VU medisch centrum
Van der Boechorststraat 7
1081 BT Amsterdam
Tel 020 4448198 (ma, di, do ochtend), 4448199 (secr.)
ah.blankenstein@vumc.nl

ERASMUS UNIVERSITEIT ROTTERDAM

ZonMW
T.a.v. Dr. Karen van Liere-Visser
Programmasecretaris VEMI
Programma Doelmatigheidsonderzoek
Projectnummer 80-82305-97-11038

Decaan Prof.dr. H.T. van der Molen
Bezoekadres Burgemeester Oudlaan 50
Postadres Postbus 1738
3000 DR Rotterdam
Doorkiesnr (010) 408 2065
Fax (010) 408 9103
E-mail decaan@fsw.eur.nl
Internet www.eur.nl/fsw

Ons kenmerk
HvdM/fsw/14541/tg

Uw kenmerk

Datum
1 februari 2010

Onderwerp
Bevestiging deelname onderzoek

Beste mevrouw Van Liere,

De Faculteit Sociale Wetenschappen van de Erasmus Universiteit Rotterdam vindt het van belang om samen met het Erasmus MC onderzoek te doen.

Ondergetekenden ondersteunen de uitgewerkte subsidieaanvraag voor onderzoek naar "Effectiviteit en doelmatigheid van een training consultvaardigheden voor medisch specialisten gericht op patiënten met somatisch onvoldoende verklaarde klachten (SOLK)".

Ondergetekenden zijn actief in de vorm van inhoudelijke en methodologische begeleiding van het onderzoek vanuit hun eigen expertise op het gebied van effectiviteit en doelmatigheid.

Met hartelijke groet,

Prof Dr H.T. van der Molen,
hoogleraar psychologie en decaan
Faculteit der Sociale Wetenschappen

Dr. L..R. Arends,

Biostatisticus,
Instituut voor Psychologie, FSW

Erasmus MC
Universitair Medisch Centrum Rotterdam

Dienst Psychosociale Zorg

ZonMW
T.a.v. Dr. Karen van Liere-Visser
Programmasecretaris VEMI
Programma Doelmatigheidsonderzoek
Projectnummer 80-82305-97-11038

Doorkiesnummer 010-703 22 33
Faxnummer 010-703 42 48
Kamernummer V-200
E-mail a.weiland@erasmusmc.nl
Ons kenmerk AW
Datum 1 februari 2010

Postadres
Postbus 2040
3000 CA Rotterdam

Bezoekadres
's Gravendijkwal 230
3015 CE Rotterdam

Parkeergarage
Westzeedijk 361
3015 AA Rotterdam

Betreft Bevestiging deelname onderzoek

Beste mevrouw Van Liere,

Het NIVEL vindt het van belang om samen met het Erasmus MC onderzoek te doen naar de "Effectiviteit en doelmatigheid van een training consultvaardigheden voor medisch specialisten gericht op patiënten met somatisch onvoldoende verklaarde klachten (SOLK)".

Ondergetekende ondersteunt de uitgewerkte subsidieaanvraag en participeert actief in de inhoudelijke begeleiding van dit onderzoek vanuit de expertise van het NIVEL als projectadviseur op het gebied van onderzoek naar SOLK en arts-patiënt communicatie.

Met hartelijke groet,

Dr. A. M. van Dulmen

ACADEMIE VOOR
MEDISCH SPECIALISTEN

Postbus 8153 • 3503 RD Utrecht
Orteliuslaan 750 • 3528 BB Utrecht
Telefoon (030) 247 4197
info@academiemedischspecialisten.nl
www.academiemedischspecialisten.nl

Erasmus Medisch Centrum
t.a.v. mevrouw drs. A. Weiland, sectormanager
Kamer V-200
Postbus 2040
3000 CA ROTTERDAM

Utrecht	: 26 januari 2010
Behandeld door	: Anita van Amerongen
Onze referentie	: ava/2010/docenten
E-mail	a.van.amerongen@academiems.nl
Betreft	: training consultvaardigheden

Beste Anne,

Naar aanleiding van ons gesprek op 19 januari j.l. bevestig ik bij dezen, dat ik geïnteresseerd ben in het landelijk aanbieden van de training consultvaardigheden voor medisch specialisten, gericht op patiënten met somatisch onvoldoende verklaarde klachten.

Zoals ik je vertelde is de Academie voor Medisch Specialisten een samenwerkingsverband van de Orde van Medisch Specialisten, de VvAA en het instituut Beleid & Management Gezondheidszorg van de Erasmus Universiteit Rotterdam. Wij verzorgen disciplineoverstijgende scholing voor medisch specialisten, overige leden van medische staven en aios. Deze training zou prima passen in ons aanbod. Wij zullen die dan ook mee gaan nemen in onze algemene werving. De feitelijke disseminatie zal uiteindelijk uiteraard afhankelijk zijn van de mate, waarin medisch specialisten bereid zijn om zich voor deze training in te schrijven en er voor te betalen.

Met vriendelijke groet,

drs. P.Wijnsma,
directeur

FLOW CHART

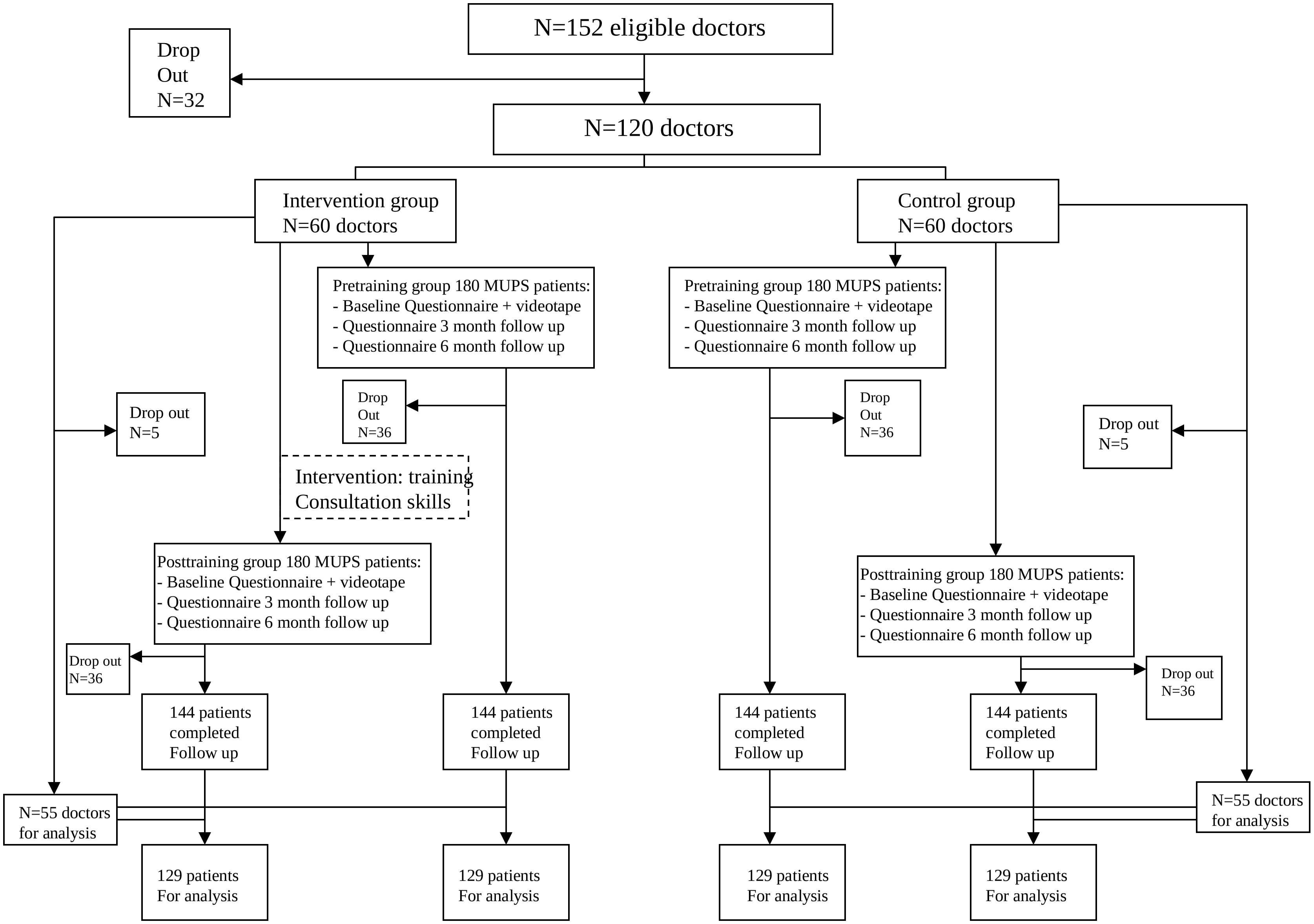
N=152 eligible doctors
Drop Out N=32
N=120 doctors
Intervention group N=60 doctors
Control group N=60 doctors
Pretraining group 180 MUPS patients:
- Baseline Questionnaire + videotape
- Questionnaire 3 month follow up
- Questionnaire 6 month follow up
Drop Out N=36
Drop out N=5
Intervention: training Consultation skills
Posttraining group 180 MUPS patients:
- Baseline Questionnaire + videotape
- Questionnaire 3 month follow up
- Questionnaire 6 month follow up
Drop out N=36
144 patients completed Follow up
N=55 doctors for analysis
129 patients For analysis

Time schedule measurements

months	1	2	3	4	5	6	7	8	9	10	11	12	13	14	15
intervention group				12 doctors selecting each 3 patients on video-Tape						selection patients on video-Tape post training					
				36 patients Baseline Q Pretraining group			Q 3 months follow up Pretraining group			Q 6 months follow up Pretraining group					
										36 patients Baseline Q Posttraining group			Q 3 months follow up Posttraining group		
control group				12 doctors selecting each 3 patients on video-Tape						selection patients on video-Tape post training					
				36 patients Baseline Q Pretraining group			Q 3 months follow up Pretraining group			Q 6 months follow up Pretraining group					
										36 patients Baseline Q Posttraining group			Q 3 months follow up Posttraining group		

Time schedule measurements

16
Q 6 months follow up Posttraining group
Q 6 months follow up Posttraining group

Begroting voor subsidie-aanvragen ZonMw
Wetenschappelijke instellingen

Dossiernummer Project:
Titel Project:
Aantal maanden looptijd Project:

Alvorens u deze begroting invult, verzoeken wij u kennis te nemen van de Subsidievoorwaarden ZonMw, in het bijzonder bijlage 2.

De geldende tabelbedragen conform het "Akkoord bekostiging wetenschappelijk onderzoek 2008" zijn te vinden op de website van ZonMw : www.zonmw.nl/............

1. Personeel

**Soort aanstelling/functie**	**Aantal maanden**	**Tabelbedrag** *(onder "afrekening indien aantal maanden")*	**Fte (%)**	**Totaal (€)**
Sen. Wet Medewerker	24	€ 127.624,00	60%	€ 76.574,40
Sen. Wet Medewerker	24	€ 127.624,00	20%	€ 25.524,80
Sen. Wet Medewerker	24	€ 127.624,00	10%	€ 12.762,40
NWP MBO	24	€ 96.307,00	10%	€ 9.630,70
NWP MBO	24	€ 96.307,00	20%	€ 19.261,40
NWP MBO	24	€ 96.307,00	15%	€ 14.446,05
NWP MBO	24	€ 96.307,00	45%	€ 43.338,15
				€ -
				€ -
**Totaal**				**€ 201.537,90**

Er is een onderscheid tussen VSNU instellingen (o.a. Universiteiten) en NFU instellingen (o.a. UMC's).
Voor ***VSNU*** *instellingen gelden de volgende functies: Promovendi, Senior wetenschappelijk medewerker, Niet-wetenschappelijk personeel MBO, Niet-wetenschappelijk personeel HBO en Niet--wetenschappelijk personeel Academisch.*
Voor ***NFU*** *instellingen gelden de volgende functies: Promovendi, PostDoc, (Arts)onderzoeker, Niet-*

Wetenschappelijk medewerker MBO, Niet-wetenschappelijk medewerker HBO en Niet-wetenschappelijk medewerker Academisch.
De per functie geldende salaristabellen voor VSNU en NFU instellingen vindt u op onze website www.zonmw.nl.

2. Persoonsgebonden benchfee (per aanstelling van wetenschappelijk personeel conform art.2.2 van bijlage 2, behorend bij de ZonMw subsidievoorwaarden)

**Omschrijving**	**Totaal (€)**
Persoonsgebonden benchfee	€ 5.000,00
**Totaal**	**€ 5.000,00**

3. Materieel, Apparatuur, Verbruiksgoederen (gespecificeerd)

**Omschrijving**	**Totaal (€)**
Video opname apparatuur + 2 laptop veldwerk	€ 7.000,00
Zaalhuur training inclusief apparatuur	€ 7.200,00
Cursus materiaal + folders	€ 2.700,00
Train de trainercursus	€ 6.600,00
Trainingen interventiegroep	€ 55.000,00
**Totaal**	**€ 78.500,00**

4. Implementatiekosten (gespecificeerd)

**Omschrijving**	**Totaal (€)**
Publications	€ 3.000,00
patienten informatie	€ 10.000,00
Supervisie	€ 5.000,00
Training specialisten in de rotterdamse regio	€ 30.000,00
**Totaal**	**€ 48.000,00**

5. Bijdragen van eigen instelling en derden

**Omschrijving**	**Totaal (€)**
	€ 130.000,00
	€ 80.000,00
**Totaal**	**€ 210.000,00**

ZonMw budget

Kostenpost	Totaal (€)
1. Personeel	€ 201.537,90
2. Persoonsgebonden benchfee (per aanstelling cf. Subsidievoorwaarden)	€ 5.000,00
3. Materieel, Apparatuur, Verbruiksgoederen (gespecificeerd)	€ 78.500,00
4. Implementatiekosten (gespecificeerd)	€ 48.000,00
Totale lasten	€ 333.037,90
Minus:	
5. Bijdragen van eigen instelling c.q. derden	€ 210.000,00
**Aan te vragen subsidie bij ZonMw**	**€ 123.037,90**

Akkoord Hoofdaanvrager:

Naam: Prof. dr. J.L.C.M. van Saase
Functie: **Projectleider**

Datum: 01-02-2010

Akkoord Financieel verantwoordelijke ontvangende instelling:

Naam: Radjan Biharie
Functie: Adviseur Financien
Telefoonnummer: 010-7035505
E-mailadres: r.biharie@erasmusmc.nl

Inclusion schedule doctors and patients

study month	1	2	3	4	5	6	7	8	9	10	11	12	13	14	15	16	17
intervention group 1			12 spec	36 pat Ipre						36 pat Ipost							
control group 1			12 spec	36 pat Cpre						36 pat Cpost							
intervention group 2				12 spec	36 pat Ipre						36 pat Ipost						
control group 2				12 spec	36 pat Cpre						36 pat Cpost						
intervention group 3					12 spec	36 pat Ipre						36 pat Ipost					
control group 3					12 spec	36 pat Cpre						36 pat Cpost					
intervention group 4						12 spec	36 pat Ipre						36 pat Ipost				
control group 4						12 spec	36 pat Cpre						36 pat Cpost				
intervention group 5							12 spec	36 pat Ipre						36 pat Ipost			
control group 5							12 spec	36 pat Cpre						36 pat Cpost			

PATIENTENINFORMATIE IN WACHTKAMER

Het spreekuur van dokter …………zal vandaag worden opgenomen op videoband ivm onderzoek en opleiding. Wanneer u hier bezwaar tegen heeft kunt u dat tegen de dokter zeggen.
In dat geval wordt de opname niet gemaakt.

PATIENTENINFORMATIE FOLDER

TITEL "Gespreksvoering voor specialisten"

Waarom dit onderzoek?

Een belangrijk instrument in het werk van de medisch specialist is de gespreksvoering met de patiënt. Hoe vaardiger een specialist daarin is, des te beter weet hij met u de passende toon te treffen. Bij ruim 30% van de patiënten, die verwezen wordt naar een medisch specialist voor nader onderzoek van hun lichamelijk klachten, kan er geen voldoende verklaring gevonden worden. Deze onduidelijkheid in de diagnose leidt heel gemakkelijk tot misverstanden in de communicatie tussen arts en patiënt. Patiënten voelen zich soms niet serieus genomen en dokters weten niet goed welke informatie en behandeling te geven als er geen medische diagnose is vastgesteld.

Daarom is er een training gespreksvoering ontwikkeld voor medisch specialisten, gericht op patiënten met klachten, die niet meteen of maar gedeeltelijk verklaarbaar zijn. Met onderzoek in de praktijk willen we duidelijk krijgen of de training, die ontwikkeld is ook effect heeft. En daar hebben we uw hulp bij nodig!

Wat houdt deelname aan het onderzoek voor u als patiënt in?

Wanneer u accoord gaat met deelname aan het onderzoek vragen we u:

- Toestemming om de video-opname van uw consult te gebruiken voor onderzoek.
- Een vragenlijst in te vullen op 3 momenten (meteen na afloop van dit consult, na 3 maanden en na een half jaar). Deze vragenlijst bevat vragen over uw gezondheid, hoe uw klachten uw leven beïnvloeden en hoe tevreden u bent over het contact met uw specialist.
- Het ingevulde toestemmingsformulier voor deelname aan het onderzoek terug te sturen aan ons.

Vertrouwelijkheid van gegevens

We behandelen al uw gegevens vertrouwelijk. Dat betekent dat we uw naam, geboortedatum en adres niet in de administratie van het onderzoek invoeren. Alle informatie verwerken we anoniem. Uw naam zal niet in latere publicaties terug te vinden zijn.

Vrijwilligheid en toestemming

We hopen dat u mee wilt werken aan dit onderzoek. Deelname is op vrijwillige basis. Als u besluit tot medewerking is het van belang dat u daar schriftelijk toestemming voor verleent door ondertekening van bijgaand 'toestemmingsfomulier'.

Indien u afziet van deelname heeft dit uiteraard geen nadelige gevolgen voor de behandeling bij uw specialist. U kunt zich ook op een later tijdstip nog terugtrekken, zonder dat dit enige gevolgen heeft voor uw behandeling.

Verzekering (vooruitlopend op positieve beoordeling)

Aangezien deelnemen aan dit onderzoek geen risico's met zich mee brengt, heeft de Medisch Ethische Toetsings Commissie van het Erasmus MC , ontheffing verleend van de verplichting tbv de deelnemers een speciale schadeverzekering af te sluiten.

Meer informatie en contact
Mocht u na het lezen van deze brief nog vragen hebben of aanvullende informatie ontvangen, dan kunt u op werkdagen contact opnemen met één van de onderzoekers.
Ook kunt u zich met vragen of voor advies over deelname wenden tot een onafhankelijke arts: Hans Faddegon (huisarts), 010 – 7043505

Hartelijke groet en bij voorbaat dank voor uw medewerking,

Anne Weiland en Nettie Blankenstein

Anne Weiland
Onderzoeker
Erasmus MC
Afdeling Inwendige Geneeskunde
Tel 010 – 7032233
Mob 06-18559690
a.weiland@erasmusmc.nl

Nettie Blankenstein
Huisarts/Senior-onderzoeker
EMGO+ instituut
Afdeling Huisartsgeneeskunde
Tel 020 – 4448198 (ma/do)
ah.blankenstein@vumc.nl

ZonMW
T.a.v. Dr. Karen van Liere-Visser
Programmasecretaris VEMI
Programma Doelmatigheidsonderzoek
Projectnummer 80-82305-97-11038

Doorkiesnummer 010 - 703 4681
Faxnummer 010 - 703 4682
Kamernummer Ba-391
E-mail e.j.kuipers@erasmusmc.nl
Ons kenmerk 106/ek/wh
Datum 2 februari 2010

Postadres
Postbus 2040
3000 CA Rotterdam

Bezoekadres
's Gravendijkwal 230
3015 CE Rotterdam

Parkeergarage
Westzeedijk 361
3015 AA Rotterdam

Prof.dr. E.J. Kuipers
Afdelingshoofd

Dr. T.J.M. van der Cammen
Sectorhoofd Geriatrie
Prof.dr. R. Gerth van Wijk
Sectorhoofd Allergologie
Dr. P.M. van Hagen
Sectorhoofd Immunologie
Prof.dr. A.J. van der Lelij
Sectorhoofd Endocrinologie
Dr. J.L. Nouwen
Sectorhoofd Infectieziekten
Dr. E.J.G. Sijbrands
Sectorhoofd Farmacologie,
Vasculaire en Metabole Ziekten
Prof.dr. W. Weimar
Sectorhoofd Niertransplantatie
Dr. R. Zietse
Sectorhoofd Nefrologie

Prof.dr. J.L.C.M. van Saase
Opleider Inwendige Geneeskunde

Betreft Reactie op advies subcommissie

Beste mevrouw Van Liere,

Allereerst willen we u danken voor het positieve advies en het commentaar van de subcommissie betreffende ons projectidee “Effectiviteit en doelmatigheid van een training consultvaardigheden voor medisch specialisten gericht op patiënten met somatisch onvoldoende verklaarde klachten (SOLK)”.

In de definitieve subsidieaanvraag hebben we uw vraag om meer informatie over het vooronderzoek ter onderbouwing van de werkzaamheid van de te onderzoeken interventie, in dit project een training consultvaardigheden voor medisch specialisten, als volgt uitgewerkt:

1. In het “Plan van aanpak” onder ‘preliminary studies’ hebben we eigen vooronderzoek beschreven evenals de opzet en resultaten van de pilottraining. In de ‘systematic review’ hebben we een RCT besproken over het belang van effectieve geruststelling bij SOLK-patiënten.
2. Daarnaast hebben we in “Probleemstelling”en “Relevantie” aanvullend onderzoek verwerkt, dat onzes inziens de werkzaamheid van de te onderzoeken interventie ondersteunt.
3. Ter versterking van het onderzoeksteam op de vragen naar effectiviteit en doelmatigheid van de interventie hebben we prof. dr. Henk van der Molen, ervaren onderzoeker naar effectiviteit van vaardigheidstrainingen en dr. Lidia Arends, als biostatisticus ervaren met onderzoek naar doelmatigheid en kosteneffectiviteit bereid gevonden met ons hierin samen te werken.

Met deze informatie over het vooronderzoek ter onderbouwing van de werkzaamheid van de interventie hopen wij voldaan te hebben aan de voorwaarden om voor honorering van subsidie in aanmerking te komen.

Met hartelijke groet,

Prof.dr. E.J. Kuipers
mede namens Prof.dr. J.C.L..M. van Saase
Drs. A. Weiland
